# NELFA and BCL2 induce the 2C‐like state in mouse embryonic stem cells in a chemically defined medium

**DOI:** 10.1111/cpr.13534

**Published:** 2023-08-17

**Authors:** Baojiang Wu, Yanqiu Wang, Xinhua Wei, Jingcheng Zhang, Jiahui Wu, Guifang Cao, Yong Zhang, Jun Liu, Xihe Li, Siqin Bao

**Affiliations:** ^1^ The State Key Laboratory of Reproductive Regulation and Breeding of Grassland Livestock Inner Mongolia University Hohhot China; ^2^ Research Centre for Animal Genetic Resources of Mongolia Plateau, College of Life Sciences Inner Mongolia University Hohhot China; ^3^ Key Laboratory of Animal Biotechnology of the Ministry of Agriculture and Rural Affairs, College of Veterinary Medicine Northwest A&F University Yangling China; ^4^ School of Veterinary Medicine Inner Mongolia Agricultural University Hohhot China; ^5^ Inner Mongolia Saikexing Institute of Breeding and Reproductive Biotechnology in Domestic Animal Hohhot China

## Abstract

A minority of mouse embryonic stem cells (ESCs) display totipotent features resembling 2‐cell stage embryos and are known as 2‐cell‐like (2C‐like) cells. However, how ESCs transit into this 2C‐like state remains largely unknown. Here, we report that the overexpression of negative elongation factor A (*Nelfa*), a maternally provided factor, enhances the conversion of ESCs into 2C‐like cells in chemically defined conditions, while the deletion of endogenous *Nelfa* does not block this transition. We also demonstrate that *Nelfa* overexpression significantly enhances somatic cell reprogramming efficiency. Interestingly, we found that the co‐overexpression of *Nelfa* and *Bcl2* robustly activates the 2C‐like state in ESCs and endows the cells with dual cell fate potential. We further demonstrate that *Bcl2* overexpression upregulates endogenous *Nelfa* expression and can induce the 2C‐like state in ESCs even in the absence of *Nelfa*. Our findings highlight the importance of BCL2 in the regulation of the 2C‐like state and provide insights into the mechanism underlying the roles of *Nelfa* and *Bcl2* in the establishment and regulation of the totipotent state in mouse ESCs.

## Graphical abstract

In mouse embryonic stem cells (ESCs), a minority of cell population displays totipotent features resembling 2‐cell‐stage embryos, known as 2‐cell‐like (2C‐like) cells. However, how ESCs transit into the 2C‐like state remains largely unknown. Here, we report that overexpression of maternal factor‐negative elongation factor A (*Nelfa*) increases the conversion of the 2C‐like state in ESCs in chemically defined condition while deletion of endogenous *Nelfa* did not block the transition of 2C‐like state. We also demonstrate that overexpression of *Nelfa* significantly increases the efficiency of somatic cell reprogramming. Interestingly, overexpression of *Nelfa* combined with *Bcl2* robus activates the 2C‐like state in ESCs which exhibit the ability of bi‐potential cell fate. We further demonstrate that overexpression of *Bcl2* increases endogenous *Nelfa* expression and can successfully induce the 2C‐like state of ESCs even in the absence of *Nelfa*. Our findings highlight an important role of BCL2 in 2C‐like state and provide insights into the mechanism underlying *Nelfa* and *Bcl2* mediated totipotent state in mouse ESCs.

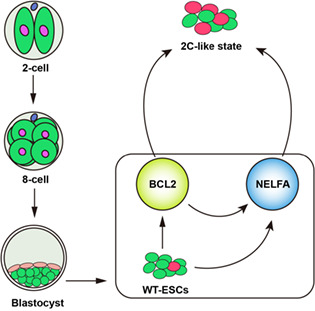

## 1 INTRODUCTION

In mice, the zygote and both blastomeres of the two‐cell (2C) stage embryo exhibit totipotency and can give rise to all embryonic and extraembryonic cells.^1^ Totipotency is a transient feature that is gradually lost as development progresses.^2^ The blastocyst consists of three cell lineages, namely, the inner cell mass (ICM), the primitive endoderm and the trophectoderm (TE). Embryonic stem cells (ESCs), trophoblast stem cells and extraembryonic endoderm cells can all be derived from blastocysts.^3^, ^4^, ^5^ Furthermore, different types of stem cells have been obtained from preimplantation embryos at different developmental stages, such as expanded potential stem cells,^6^ totipotent‐like stem cells and totipotent potential stem cells.^7^, ^8^ Establishing these different types of stem cell lines provides a platform for studying the mechanisms involved in the conversion between different stem cell states.

Macfarlan et al. found that a small subpopulation of ESCs displays features similar to those found in the 2C embryo.^9^ These 2C‐like cells have similar transcriptional profiles to 2C embryos,^10^, ^11^ and thus represent a good model for investigating the conversion of the pluripotent to the totipotent state and zygotic genome activation (ZGA). Multiple strategies have been reported for converting ESCs into 2C‐like cells, including the overexpression of 2C‐like genes,^12^, ^13^ ‘active’ histone modification,^9^ DNA hypomethylation,^12^, ^14^, ^15^ chromatin remodelling,^7^, ^16^, ^17^ chemically induced double‐stranded DNA breaks,^18^, ^19^, ^20^ cell cycle regulation,^21^, ^22^ and metabolic reprogramming.^13^, ^23^ In addition, studies have shown that the strategies used for the induction of 2C‐like cells from ESCs also improve the efficiency of somatic cell nuclear transfer (SCNT) and the reprogramming of somatic cells into induced pluripotent stem cells (iPSCs).^24^, ^25^, ^26^ Most media used for the induction of the 2C‐like state contain serum. However, the composition of serum is complex, and it is still unclear, which constituents play a key role in inducing the 2C‐like state. Additionally, it has been reported that when a chemically defined naïve culture medium (N2B27 medium containing CHIR99021, PD0325901 and LIF; also known as 2i/L) was used, the pluripotent to totipotent state conversion could not be achieved.^13^, ^27^

We have previously shown that the maternal factor negative elongation factor A (NELFA) promotes the transformation of mouse ESCs into 2C‐like cells in serum‐containing medium and that the *Nelfa*/*Dux*/*Zscan4* axis plays a regulatory role during this transformation.^13^ We also demonstrated that retinoic acid (RA) treatment activates the NELFA‐mediated 2C‐like state in mouse ESCs and increases the 2C‐like cell population in culture in the absence of serum or genetic modification.^28^ Moreover, studies have also shown that *Dux* overexpression can induce the 2C‐like state in mouse ESCs.^29^, ^30^, ^31^ Nevertheless, this effect is accompanied by significant cell apoptosis, making it difficult to maintain the self‐renewal of 2C‐like cells.^32^ In addition, human ESCs that overexpress the anti‐apoptotic genes B cell leukaemia/lymphoma 2 (*BCL2*) or *BCL2L1* effectively contribute to interspecific human–mouse chimeras when human ESCs are injected into mouse embryos.^33^, ^34^, ^35^ Accordingly, we asked whether anti‐apoptotic genes contribute to the self‐renewal potential of 2C‐like cells through anti‐apoptotic mechanisms. In this study, we found that the overexpression of *Bcl2* alone or in combination with *Nelfa* could robustly activate the 2C‐like state in ESCs. Interestingly, several strategies utilised for the induction of 2C‐like cells from ESCs were found to also enhance the efficiency of SCNT as well as the reprogramming of somatic cells into iPSCs.^24^, ^25^, ^26^ Here, we found that *Nelfa* overexpression together with Yamanaka's four factors can enhance somatic cell reprogramming efficiency. Recently, Liuyang et al. established a highly efficient and rapid chemical reprogramming system for converting human somatic cells into iPSCs,^36^, ^37^ emphasizing the importance of a chemically defined stem cell culture system in vitro. Given that chemically defined conditions provide a robust approach for cell fate manipulation, the induction of 2C‐like cells using chemically defined media may provide valuable in vitro models for dissecting common regulators or pathways involved in pluripotency and early embryonic development.

## 2 MATERIALS AND METHODS

### 2.1 Animals

All procedures involving animals were conducted following the guidelines of Inner Mongolia University, China. Mice were housed in a temperature‐controlled room with proper darkness‐light cycles, fed with a regular diet and maintained under the care of the Laboratory Animal Unit, Inner Mongolia University, China. The mice were sacrificed by cervical dislocation. This study was approved by the Institutional Animal Care and Use Committee of Inner Mongolia University, China. Oct4‐ΔPE‐GFP (GOF/GFP) transgenic mice in a mixed background (MF1, 129/sv and C57BL/6J strains) were used for generating ESC lines.^38^ ICR mice were used in chimeric embryo experiments. For superovulation, ICR female mice (6–8 weeks old) were intraperitoneally injected with 5 IU of pregnant mare serum gonadotropin (PMSG, Ningbo Sansheng, 110,915,564) and then with human chorionic gonadotropin (hCG, Ningbo Sansheng, 110,911,282) after 48 h, and then mated overnight. Eight‐cell stage embryos were collected 72 h after hCG injection.

### 2.2 Cell lines

Mouse ESCs were obtained as previously reported^39^, ^40^, ^41^ and maintained in 2i/L medium (1000 IU/mL LIF [Millipore, ESG1107], 1 μM PD0325901 [Miltenyi Biotec, 130–103‐923] and 3 μM CHIR99021 [Miltenyi Biotec, 130–103‐926] in N2B27 basal medium) on fibronectin‐coated (Millipore, FC010) plates. N2B27 basal medium contained one volume of DMEM/F12 (Gibco, 11,320–033) combined with one volume of neurobasal medium (Gibco, 21,103–049) supplemented with 0.5% N2 supplement (Gibco, 17,502–048), 1% B27 supplement (Gibco, 17,504–044), 2 mM GlutaMAX‐l (Gibco, 35,050–061), 1% MEM NEAA (Gibco, 11,140–050), 1% penicillin/streptomycin (Gibco, 15,140–122), 50 mg/L bovine serum albumin (Gibco, 15,260–037) and 110 μM β‐mercaptoethanol (Sigma, M3148).

### 2.3 *Nelfa* knockout (KO) vector construction and transfection

For CRISPR‐Cas9‐mediated *Nelfa* KO, guide RNA (gRNA) oligonucleotides were designed using the online CRISPR design tool (https://zlab.bio/guide-design-resources) and those least likely to have off‐target effects based on software prediction were selected. gRNAs were annealed with their respective reverse oligonucleotides, cloned into the PX459 plasmid and transformed into DH5α cells (Takara, 9057). Following preliminary electrophoresis‐based screening, potential *Nelfa* KO plasmid were sent for sequencing using the primer 5′‐GGGCCTATTTCCCATGATTCCT‐3′. Following the manufacturer's instructions, ESCs were co‐transfected with three pairs of gRNA and enhanced green fluorescent protein (EGFP) using Lipofectamine 2000 (Invitrogen, 11,668–027) and selected with puromycin (MedChemExpress, HY‐15695) for 3–5 days. Genomic DNA was extracted from single cell‐derived colonies after puromycin screening and genotyped to establish the *Nelfa* KO ESCs. The gRNA sequences used are given in Table S1.

### 2.4 *Nelfa* and/or *Bcl2* overexpression vector construction and ESC transfection

cDNA encoding *Nelfa* and *Bcl2* was PCR amplified from mouse cDNA and cloned either separately or in combination into the piggyBac‐based doxycycline (Dox)‐inducible vector upstream of mCherry. For the establishment of stem cell lines with the piggyBac‐based Tet‐on inducible overexpression system, ESCs were transfected with Tet‐on‐TRE‐*Nelfa*‐mCherry (2 μg), Tet‐on‐TRE‐*Bcl2*‐mCherry (2 μg), or Tet‐on‐TRE‐*Nelfa*‐*Bcl2*‐mCherry (2 μg) together with the piggyBac transposase‐expressing vector (PB‐EF1α‐transposase) (2 μg) and PB‐EF1α‐rtTA plasmids using Lipofectamine 2000. After 3 days of transfection, mCherry‐positive cells were sorted via fluorescence‐activated cell sorting (FACS) and then seeded into 24‐well plates (Corning, 3524).

### 2.5 Flow cytometry

ESCs expressing the mCherry and EGFP reporters were harvested and sorted (568 and 488 nm, respectively) using the BD FACSAria Sorp cell sorter. ESCs lacking mCherry or EGFP were used as negative controls. Residual cell debris was gated out and diploid and tetraploid DNA peaks were measured. A region representing mCherry‐positive or EGFP‐positive cells was used to identify living cells.

### 2.6 Mouse iPSC generation

To generate iPSCs, GOF/GFP‐carrying transgenic mouse embryo fibroblasts (MEF^GOF/GFP^) were co‐transfected with Tet‐on‐TRE‐*Nelfa*‐mCherry (2 μg) and PB‐EF1α‐transposase (2 μg), PB‐EF1α‐rtTA and PB‐TRE‐h4F (human *OCT4*, *cMYC*, *SOX2* and *KLF4*) (2 μg). The transfected cells (2 × 10^5^) were seeded into feeder layer‐coated 24‐well plates and cultured in KO DMEM (Gibco, 10,829–018) containing 10% KO serum replacement (Gibco, 10,828–028), 10% fetal bovine serum (FBS; Gibco, 10,099–141), 2 mM GlutaMAX‐l, 1% MEM NEAA, 1% penicillin/streptomycin, 110 μM β‐mercaptoethanol, 3 μM CHIR99021, 1 μM PD0325901 and 1000 IU/mL LIF. The day after transfection, 1 μg/mL Dox (MedChemExpress, HY‐N0565) was added to the culture medium. The medium was changed every other day. Fluorescence microscopy was used to detect iPSC generation from 4 to 8 days after Dox induction. When iPSC‐like colonies were detected, they were picked up, dissociated with TrypLE (Gibco, 12,563–029), and replated in fibronectin‐coated 24‐well plates in 2i/L medium (see above).

### 2.7 Immunostaining

For immunofluorescence staining, cells or embryos were fixed for 30 min in 4% paraformaldehyde (Solarbio, P1110) at room temperature, permeabilized for 30 min in Dulbecco's phosphate‐buffered saline (DPBS; Gibco, 14,190–144) containing 0.1% Triton X‐100 (Sigma, T8787) and 1% BSA, and incubated overnight at 4°C with primary antibodies. After washing three times with DPBS containing 0.1% Triton X‐100 and 1% BSA, cells or embryos were incubated with the secondary antibody for 1 h at room temperature. The slides were then mounted in Vectashield (Sigma, P36930) with DAPI (Vector Laboratories, H‐2000) and imaged using an Olympus FV1000 confocal microscope. The primary antibodies used in this study are listed in Table S2.

### 2.8 Real‐time PCR

Total RNA was isolated using the RNeasy Mini Kit from Qiagen (74104) according to the manufacturer's protocol. cDNA was synthesized using the GoScript Reverse Transcription System (Promega, A5001). Real‐time PCR was performed with the KAPA SYBR FAST qPCR Kit (KAPA Biosystems, KK4601) on a LightCycler 96 Instrument (Roche molecular systems) with at least three biological replicates, with similar results. Relative transcript levels were assessed using the 2^−ΔΔCt^ method, with *Gapdh* serving as an endogenous control. The primer pairs used in this study are described in Table S3.

### 2.9 Western blot

Total protein was extracted using lysis buffer (Solarbio, R0030) containing 0.1 M PMSF (Solarbio, P0100) and 10 g/L phosphatase inhibitor (Thermo Scientific, A32957) on ice. After centrifugation at 13,000 × *g* for 10 min at 4°C, the protein concentration in the supernatant was determined using a Pierce BCA Protein Assay Kit (Thermo Scientific, 23,227). Protein was separated by 10%–12% SDS–PAGE, transferred to a nitrocellulose membrane (BIO‐RAD, 1620177), blocked with 5% skimmed milk (BD, 232100) for 1 h at room temperature, and then incubated first with primary antibody overnight at 4°C, and then with secondary antibody for 1 h at room temperature. The signals were visualised with Pierce ECL Western Blotting Substrate (Thermo Scientific, 32,209) and detected with the ChemiDoc Touch Imaging System (Bio‐Rad).

### 2.10 Spontaneous differentiation of ESCs

Wild‐type (WT) ESCs and *Nelfa* KO ESCs were cultured in N2B27 medium. *Nelfa*‐overexpressing ESCs (*Nelfa* OE ESCs), *Nelfa* + *Bcl2* OE ESCs and *Bcl2* OE ESCs were cultured in N2B27 medium containing Dox for 6 days. To verify whether Nelfa‐mediated 2C‐like conversion is a unique feature of pluripotency, WT ESCs and Nelfa OE ESCs were cultured in an N2B27 medium for 8 days withdrawal of Dox, followed by a 48‐h Dox re‐addition. After complete differentiation, samples were collected from WT ESCs and Dox‐treated *Nelfa* OE cells.

### 2.11 Chimeric embryo experiments

Eight‐cell stage embryos were obtained by flushing the oviducts of female ICR mice. Corresponding cells with reporter expression were dissociated and 5–10 cells were injected into each embryo. Following microinjection, 8‐cell stage embryos were cultured in KSOM (Millipore, MR‐101) for another 48 h at 37°C with 5% CO_2_. For the embryo transfer experiment, 8‐cell microinjected embryos were transferred into the oviducts of pseudopregnant females and were collected at embryonic day 6.5 (E6.5).

### 2.12 RNA sequencing (RNA‐seq) and data analysis

Total RNA was prepared using TRIzol reagent (Invitrogen, 15,596,026) according to the manufacturer's protocol. mRNA was purified from total RNA using poly‐T oligo‐attached magnetic beads. Fragmentation was performed using divalent cations under elevated temperature in 5× NEBNext first strand synthesis reaction buffer. First‐strand cDNA was synthesized using random hexamer primers and M‐MuLV reverse transcriptase. Second‐strand cDNA synthesis was performed using DNA Polymerase I and RNase H. Overhangs were converted to blunt ends via exonuclease/polymerase activity. The library fragments were purified with the AMPure XP system (Beckman Coulter). Sample clustering was performed on a cBot Cluster Generation System using the TruSeq PE Cluster Kit v3‐cBot‐HS (Illumina) according to the manufacturer's instructions. Subsequently, the library preparations were sequenced on an Illumina Novaseq platform and paired‐end reads (150 bp) were generated.

After sequencing, the paired‐end clean reads were aligned to the reference genome using Hisat2 v2.0.5. Feature Counts v1.5.0‐p3 was used to quantify the number of reads mapped to each gene. Then, the FPKM was calculated for each gene based on the gene length and read count. Differential expression analysis was performed using the DESeq2 R package (1.16.1). Gene ontology (GO) enrichment analysis of differentially expressed genes (DEGs) was implemented using the clusterProfiler R package, with correction for gene length bias. GO terms with a corrected *p*‐value <0.05 were considered significantly enriched by differentially expressed genes.

Gene set enrichment analysis (GSEA) is a computational approach for determining if a predefined gene set shows a significant, consistent difference between two biological states. The genes were ranked according to the degree of differential expression in the two samples, and then the predefined gene sets were tested to see if they were enriched at the top or bottom of the list. GSEA can include subtle expression changes. The local version of the GSEA analysis tool (http://www.broadinstitute.org/gsea/index.jsp) was used in this study. GO and KEGG data sets were subjected to GSEA independently.

### 2.13 Statistical analysis

All values are shown as means ± SD. Statistical analysis, statistical significance and *n* values are reported in the figures. Statistical analysis was performed in GraphPad Prism (version 8). The significance of differences was assessed using unpaired, two‐tailed Student's *t*‐tests. A *p*‐value <0.05 was considered significant.

## 3 RESULTS

### 3.1 *Nelfa* overexpression contributes to initiating the 2C‐like state in ESCs in 2i/L conditions

**FIGURE 1:**
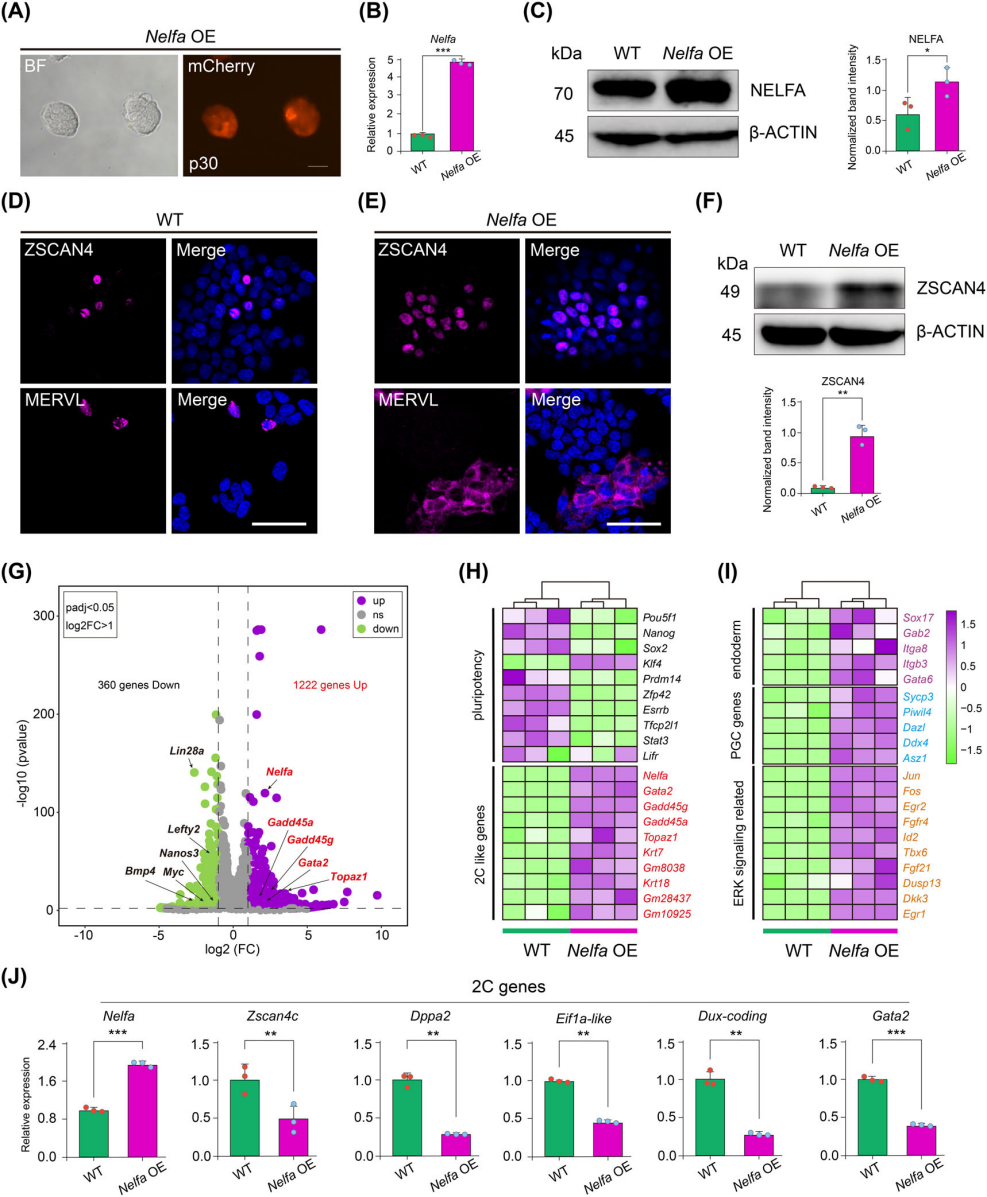
*Nelfa* partially induces the 2C‐like state in embryonic stem cells (ESCs) under chemically defined conditions. (A) Brightfield and fluorescence images showing *Nelfa* overexpressing (OE) ESCs carrying mCherry. Scale bars, 100 μm. (B) Relative expression levels of *Nelfa* in wild‐type (WT) ESCs and *Nelfa* OE ESCs as determined by RT‐qPCR. Error bars represent means ± SD (*n* = 3). The *p*‐values were calculated using two‐tailed Student's *t*‐tests, *p* < 0.05; *n* = 3 biological replicates. (C) NELFA protein levels as detected by western blot in *Nelfa* OE ESCs. β‐Actin was used as a loading control. Right: Relative NELFA expression levels calculated after normalisation to β‐actin. (D,E), Immunofluorescence staining for ZSCAN4 and MERVL in WT ESCs (D) and *Nelfa* OE ESCs (E). Cell nuclei were stained with 4′,6‐diamidino‐2‐phenylindole (DAPI). Scale bar, 50 μm. (F) ZSCAN4 protein expression levels as detected by western blot in *Nelfa* OE ESCs. β‐Actin was used as a loading control. Bottom: Relative ZSCAN4 expression levels calculated after normalisation to β‐actin. (G) Volcano plot showing differentially expressed genes (DEGs; green: downregulated, purple: upregulated) between *Nelfa* OE ESCs and WT ESCs; some of the DEGs are listed. (H) Heatmap illustrating the expression levels of pluripotency genes and 2C‐like genes in WT ESCs and *Nelfa* OE ESCs. (I) Heatmap illustrating the expression levels of genes related to the exit from naïve pluripotency in WT ESCs and *Nelfa* OE ESCs. (J) Relative expression levels of 2C genes in *Nelfa* OE ESCs versus WT ESCs after spontaneous differentiation as determined by RT‐qPCR (right). Error bars represent means ± SD (*n* = 3). The *p*‐values were calculated using two‐tailed Student's *t*‐tests, *p* < 0.05; *n* = 3 biological replicates.

Studies have shown that serum‐containing culture conditions can activate the 2C‐like state in ESCs, whereas a naïve culture condition (N2B27 medium containing CHIR99021, PD0325901 and LIF; also known as 2i/L) reportedly failed to induce a pluripotent‐to‐totipotent state conversion.^13^, ^27^ In this study, we first investigated whether *Nelfa* overexpression can induce a 2C‐like state in ESCs cultured in 2i/L. We generated ESCs carrying a Dox‐inducible *Nelfa* cDNA overexpression construct (*Nelfa* OE ESCs) with mCherry serving as the reporter (Figure S1A). After Dox induction, mCherry‐positive *Nelfa* OE ESCs were obtained by FACS (Figure 1A). Following Dox treatment, we found that *Nelfa* expression was significantly upregulated in *Nelfa* OE ESCs compared with that in WT ESCs under 2i/L culture conditions (Figure 1B,C). The overexpression of *Nelfa* in ESCs exerted no or only subtle effects on the expression of pluripotency genes (*Oct4*, *Sox2* and *Nanog*) at both the mRNA and protein levels (Figure S1B,C). To further understand the transcriptional features of *Nelfa* OE ESCs, we performed an RNA‐seq analysis on *Nelfa* OE ESCs and WT ESCs. We found that the pluripotency‐related transcriptome of *Nelfa* OE ESCs was almost identical to that of WT ESCs. Of 898 pluripotency‐specific genes previously reported,^13^ 806 were expressed in *Nelfa* OE ESCs and only 26 were differentially expressed in *Nelfa* OE ESCs relative to that seen in WT ESCs (Figure S1D), indicating that the *Nelfa* OE ESCs were in a pluripotent state.

We next explored whether *Nelfa* overexpression can induce the 2C‐like state in ESCs cultured under the 2i/L condition. Immunofluorescence staining and western blotting indicated that the protein levels of ZSCAN4 and MERVL were increased in *Nelfa* OE ESCs compared with those in WT ESCs (Figure 1D–F), indicating that the 2C‐like state had been activated in the *Nelfa* OE ESCs. In addition, our RNA‐seq data demonstrated that 1222 and 360 genes were respectively activated and repressed (log2FC >1, padj <0.05) in *Nelfa* OE ESCs compared with WT ESCs (Figure 1G). The upregulated genes included known 2‐cell embryo‐specific transcripts such as *Gata2*, *Topaz1*, *Gadd45a* and *Gadd45g* (Figure 1G). Meanwhile, the downregulated genes included pluripotency genes such as *Bmp4*, *Myc*, *Lin28a*, *Nanos3* and *Lefty2* (Figure 1G), consistent with previous reports that the activation of the 2C‐like state in *Nelfa* OE ESCs is accompanied by the downregulation of the expression of pluripotency‐related genes (Figure 1H). In *Nelfa* OE ESCs, the expression of several genes related to the exit from naïve pluripotency was significantly upregulated compared with that in WT ESCs (Figure 1I), which is consistent with studies showing that exit from naïve pluripotency is necessary for the induction of the 2C‐like state.^13^, ^42^ It was previously reported that 229 genes were upregulated in *Nelfa*‐induced cells under serum/LIF culture conditions.^13^ Here, we found that the expression of 142 genes was increased in our *Nelfa* OE ESCs under 2i/L culture conditions, but only nine showed significant upregulation (Figure S1D). These findings suggested that the overexpression of *Nelfa* partially activates the 2C‐like state under naïve culture conditions and contributes to the initial induction of the 2C‐like state in ESCs.

We also investigated whether the *Nelfa*‐mediated conversion to the 2C‐like state is unique to pluripotency or whether *Nelfa* overexpression can also induce the conversion to a 2C‐like state in differentiated cells. To this end, *Nelfa* OE ESCs were spontaneously differentiated under Dox‐free differentiation medium, following which the expression levels of pluripotency genes were measured by real‐time PCR (Figure S1E). Subsequently, the differentiated cells were treated with Dox to induce *Nelfa* overexpression. Differentiated cells overexpressing *Nelfa* did not show an increase in the expression of 2C‐associated genes (Figure 1J), suggesting that activation of the 2C‐like state induced by *Nelfa* overexpression is unique to pluripotent ESCs.

### 3.2 Endogenous *Nelfa* is dispensable for the activation of a 2C‐like state in ESCs

**FIGURE 2:**
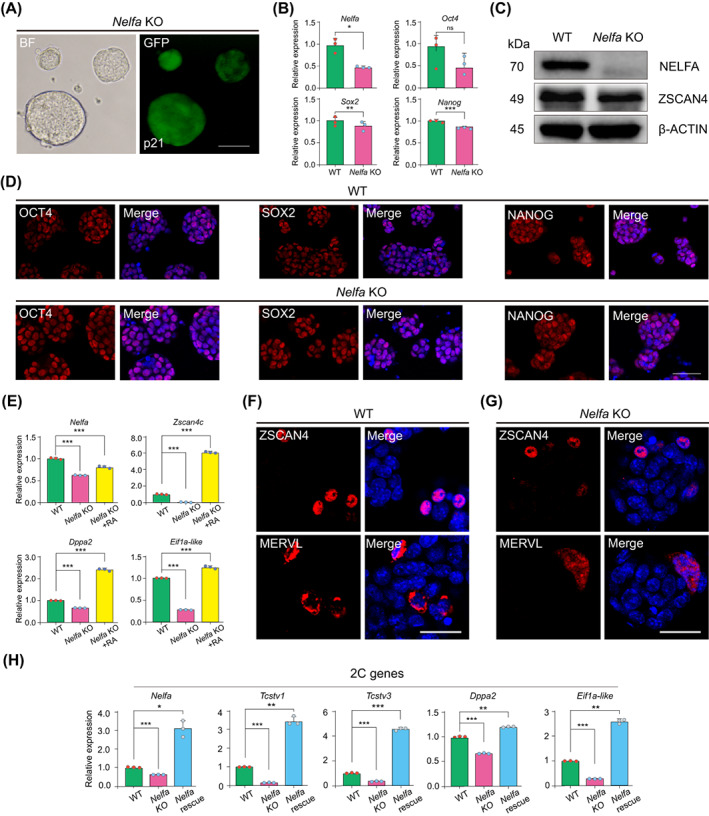
The knockout (KO) of endogenous *Nelfa* does not block the 2C‐like state in embryonic stem cells (ESCs). (A) Brightfield and fluorescence images showing EGFP‐labelled *Nelfa* KO ESCs. Scale bars, 100 μm. (B) RT‐qPCR analysis of the *Nelfa*, *Oct4*, *Sox2* and *Nanog* genes in wild‐type (WT) ESCs and *Nelfa* KO ESCs. Error bars represent means ± SD (*n* = 3). The *p*‐values were calculated using two‐tailed Student's *t*‐tests, *p* < 0.05; *n* = 3 biological replicates. (C) Western blot of the indicated proteins in WT ESCs and *Nelfa* KO ESCs. β‐Actin was used as a loading control. (D) Immunofluorescence staining for OCT4, SOX2 and NANOG in WT ESCs (top) and *Nelfa* KO ESCs (bottom). Cell nuclei were stained with 4′,6‐diamidino‐2‐phenylindole (DAPI). Scale bar, 50 μm. (E) RT‐qPCR analysis *Nelfa*, *Zscan4c*, *Dppa2* and *Eif1a‐like* in WT ESCs, *Nelfa* KO ESCs and *Nelfa* KO + RA (retinoic acid) ESCs. Error bars represent means ± SD (*n* = 3). The *p*‐values were calculated using two‐tailed Student's *t*‐tests, *p* < 0.05; *n* = 3 biological replicates. (F,G) Immunofluorescence staining for ZSCAN4 and MERVL in WT ESCs (F) and *Nelfa* KO ESCs (G). Cell nuclei were stained with DAPI. Scale bar, 50 μm. (H) RT‐qPCR analysis of 2C‐related genes (*Nelfa*, *Tcstv1*, *Tcstv3*, *Dppa2* and *Eif1a‐lik*e) in *Nelfa* KO ESCs treated with or without doxycycline (Dox). Error bars represent means ± SD (*n* = 3). The *p*‐values were calculated using two‐tailed Student's *t*‐tests, *p* < 0.05; *n* = 3 biological replicates.

To comprehensively define the role of *Nelfa* in the activation of 2C‐like cells as well as in the pluripotency of ESCs, we established a *Nelfa* KO (*Nelfa* KO) ESCs line by CRISPR‐Cas9 co‐transfected with an EGFP (Figure 2A–C and Figure S2A). The expression of *Sox2* and *Nanog* was significantly reduced in *Nelfa* KO ESCs as well as *Oct4* expression was also decreased, but not decreased significantly (Figure 2B). In contrast, the levels of pluripotency‐related proteins were not altered (Figure 2D). Together, these data suggested that the deletion of *Nelfa* in ESCs exerts only subtle effects on ESC pluripotency. To determine whether *Nelfa* deficiency affects the differentiation ability of ESCs, we assessed the differentiation potential of *Nelfa* KO ESCs expressing an EGFP reporter in chimeric embryos by injecting 12 cells into embryos at the 8‐cell stage. We found that *Nelfa* KO ESCs contributed to the epiblast of gastrula‐stage embryos (Figure S2B,C).

To further understand the role of *Nelfa* in the activation of the 2C‐like state, we measured both the mRNA and protein levels of 2C‐like state‐related genes in *Nelfa* KO ESCs. We found that the expression levels of 2C‐related genes such as *Zscan4c*, *Dppa2* and *Eif1a‐like* were significantly lower in *Nelfa* KO ESCs than in WT ESCs (Figure 2E). Interestingly, no differences in ZSCAN4 and MERVL protein levels were observed between *Nelfa* KO and WT ESCs (Figure 2C,F,G). These data suggested that endogenous *Nelfa* is dispensable for the activation of a 2C‐like state in ESCs. Importantly, the expression levels of 2C‐related genes such as *Tcstv1*, *Tcstv3*, *Dppa2* and *Eif1a‐lik*e were rescued by the Dox‐induced exogenous overexpression of *Nelfa* in *Nelfa* KO ESCs (Figure 2H). In addition, RA treatment significantly increased the expression levels of 2C‐like genes (*Zscan4c*, *Dppa2* and *Eif1a‐like*) in *Nelfa* KO ESCs (Figure 2E). These data reinforced our finding that *Nelfa* overexpression induce the activation of a 2C‐like state in ESCs.

### 3.3 *Nelfa* combined with *Bcl2* can robustly induce the 2C‐like state in ESCs

**FIGURE 3:**
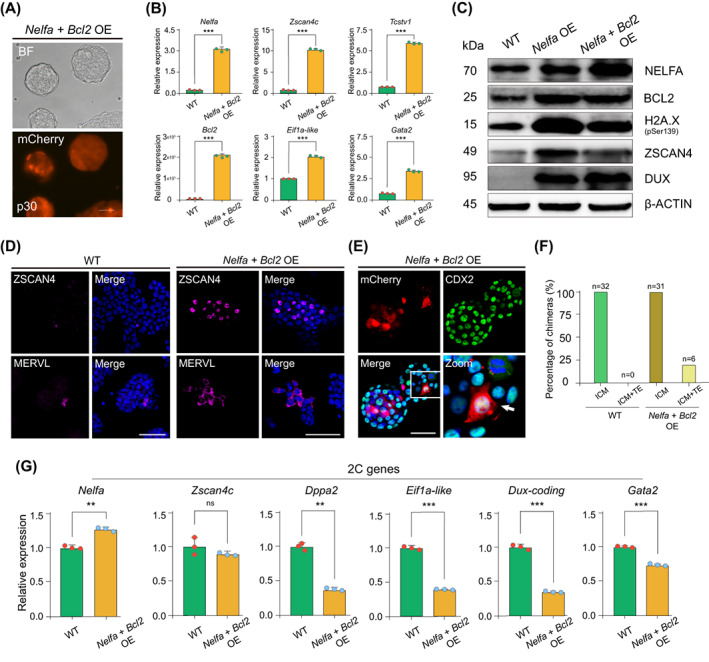
*Nelfa* combined with *Bcl2* robustly induces the 2C‐like state in embryonic stem cells (ESCs). (A) Brightfield and fluorescence images showing *Nelfa* + *Bcl2* overexpressing (OE) ESCs, with mCherry serving as a reporter. Scale bars, 100 μm. (B) RT‐qPCR analysis of the *Nelfa*, *Zscan4c*, *Tcstv1*, *Bcl2*, *Eif1a‐lik*e and *Gata2* genes in wild‐type (WT) ESCs and *Nelfa* + *Bcl2* OE ESCs. Error bars represent means ± SD (*n* = 3). The *p*‐values were calculated using two‐tailed Student's *t*‐tests, *p* < 0.05; *n* = 3 biological replicates. (C) Western blot of the indicated proteins in WT ESCs, *Nelfa* OE ESCs and *Nelfa* + *Bcl2* OE ESCs. β‐Actin was used as a loading control. (D) Immunofluorescence staining for ZSCAN4 and MERVL in WT ESCs (left) and *Nelfa* + *Bcl2* OE ESCs (right). Cell nuclei were stained with 4′,6‐diamidino‐2‐phenylindole (DAPI). Scale bar, 50 μm. (E) Representative images chimeric blastocyst immunostaining. Eight‐cell stage embryos were injected with mCherry‐labelled *Nelfa* + *Bcl2* OE ESCs and then cultured for 48 h in vitro. mCherry‐labelled *Nelfa* + *Bcl2* OE ESCs contributed to the CDX2‐positive trophectoderm (TE) cell population. Scale bars, 50 μm. (F) The summary of mCherry‐labelled *Nelfa* + *Bcl2* OE ESC‐derived E4.5 chimeras. ICM, inner cell mass; TE, trophectoderm. (G) Relative expression levels of 2C‐related genes (*Nelfa*, *Zscan4c*, *Dppa2*, *Eif1a‐lik*e, *Dux* and *Gata2*) as measured by RT‐qPCR following the spontaneous differentiation of WT ESCs and *Nelfa* + *Bcl2* OE ESCs in vitro. Error bars represent means ± SD (*n* = 3). The *p*‐values were calculated using two‐tailed Student's *t*‐tests, *p* < 0.05; *n* = 3 biological replicates.

Numerous and complex mechanisms regulate the 2C‐like state conversion in ESCs. Recently, it was shown that the *Dux*‐mediated induction of the 2C‐like state is associated with DNA damage and an increase in the levels of phosphorylated H2A.X (γH2A.X).^18^, ^19^ Because BCL2 negatively regulates the DNA damage response and DNA repair,^43^ we next asked whether the BCL2‐mediated inhibition of apoptosis and accumulation of DNA damage affected the induction of the 2C‐like state. To test this possibility, we established an ESC line carrying a construct expressing Dox‐inducible *Nelfa* and *Bcl2* cDNA (*Nelfa* + *Bcl2* OE ESCs) containing mCherry as the reporter (Figure 3A–C). Analysis of protein levels using immunofluorescence staining showed that *Nelfa* and *Bcl2* co‐overexpression did not affect OCT4, SOX2 and NANOG protein levels (Figure S3A). Interestingly, the overexpression of *Nelfa* and *Bcl2* significantly induced the expression of 2C genes such as *Zscan4c*, *Tcstv1*, *Eif1a‐like* and *Gata2* (Figure 3B). Importantly, the protein levels of NELFA, BCL2, ZSCAN4, DUX and MERVL were also increased in *Nelfa* + *Bcl2* OE ESCs compared with those in WT ESCs, as determined by immunofluorescence and western blotting (Figure 3C,D). Additionally, the level of phosphorylated H2A.X (Ser139) was significantly higher in *Nelfa* + *Bcl2* OE ESCs than in WT ESCs (Figure 3C).^44^ Interestingly, the level of phosphorylated H2A.X (Ser139) in *Nelfa* + *Bcl2* OE ESCs was intermediate between that seen in WT ESCs and *Nelfa* OE ESCs (Figure 3C). These data suggested that other regulatory mechanisms besides double‐strand breaks are involved in 2C‐like conversion in ESCs cultured in 2i/L medium.

The dual cell fate potential of early blastomeres is strictly defined by the capability of 2C‐like cells to contribute to both the ICM and the TE.^9^, ^13^ Accordingly, we microinjected mCherry‐labelled WT ESCs or *Nelfa* + *Bcl2* OE ESCs into 8‐cell embryos and analysed the fate of these cells in the resulting chimeric blastocysts. WT ESCs only contributed to the ICM of chimeric blastocysts (Figure S3B), while *Nelfa* + *Bcl2* OE ESCs colonized both the ICM and the TE (Figure 3E). Immunofluorescence staining further confirmed that, like their neighbouring ICM cells, mCherry‐positive ICM cells derived from WT ESCs strongly express OCT4 in chimeric blastocysts (Figure S3B). Additionally, whereas mCherry‐positive WT ESCs did not contribute to the CDX2‐positive TE cell population (Figure S3B), mCherry‐positive *Nelfa* + *Bcl2* OE ESCs strongly contributed to both the OCT4‐ and CDX2‐positive ICM and TE cell populations, respectively (Figure 3E,F and Figure S3C). We also found that *Nelfa* + *Bcl2* overexpression in differentiated ESCs could not induce the expression of 2C‐like genes (Figure 3G and Figure S3D), confirming that the conversion to a 2C‐like state is ESC‐specific. Our results demonstrated that *Nelfa* combined with *Bcl2* can robustly induce the 2C‐like state in ESCs.

### 3.4 The overexpression of *Nelfa* and *Bcl2* induces the 2C transcriptional programme in ESCs

**FIGURE 4:**
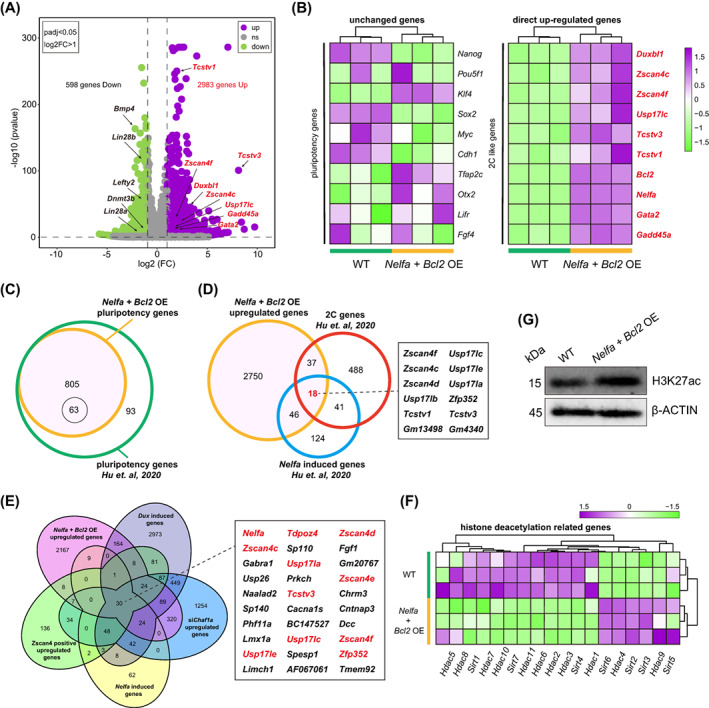
The overexpression of *Nelfa* and *Bcl2* induces the 2C transcriptional programme in embryonic stem cells (ESCs). (A) Volcano plot showing differentially expressed genes (DEGs; green: downregulated, purple: upregulated) between *Nelfa* + *Bcl2* overexpressing (OE) ESCs and wild‐type (WT) ESCs; some DEGs are listed. (B) A heatmap showing the expression levels of pluripotency genes (left) and 2C‐like genes (right) in *Nelfa* + *Bcl2* OE ESCs and WT ESCs. (C) Venn diagram showing that of the 898 genes previously reported as being pluripotency‐specific, 805 were also expressed in *Nelfa* + *Bcl2* OE ESCs. (D) A Venn diagram showing that 64 *Nelfa*‐induced genes and 55 two‐cell embryo‐specific genes were upregulated in *Nelfa* + *Bcl2* OE ESCs (e.g., *Zscan4f*, *Zscan4c*, *Zscan4d*, *Zfp352* and *Usp17lc*). (E) Venn diagram showed that 30 genes were overlapped in *Nelfa* + *Bcl2* OE ESCs upregulated genes compared with previously published *Zscan4* positive upregulated, *Dux* induced, *Chaf1a* depletion and *Nelfa* induced genes, such as *Nelfa*, *Tdpoz4*, *Zscan4d*, *Zscan4c* and *Usp17la*. (F) A heatmap showing the expression levels of histone deacetylation‐related genes in *Nelfa* + *Bcl2* OE ESCs and WT ESCs. (G) The protein levels of acetylated H3 (H3K27ac) in *Nelfa* + *Bcl2* OE ESCs and WT ESCs as detected by western blot. β‐Actin served as a loading control.

Given that the above results indicated that *Nelfa* + *Bcl2* OE ESCs exhibit the properties of a 2C‐like state, we next asked whether these cells display a fully activated 2C‐like cell transcriptome. We compared the transcriptomes of *Nelfa* + *Bcl2* OE ESCs and WT ESCs (the original pluripotent cells) and identified 2983 upregulated and 598 downregulated genes (log2FC >1, padj <0.05) in *Nelfa* + *Bcl2* OE ESCs (Figure 4A). The upregulated genes included those enriched in the GO terms regulation of transsynaptic signalling, modulation of chemical synaptic transmission and extracellular structure organisation; meanwhile, the downregulated genes were enriched in the response to interferon‐gamma, neural crest cell development and stem cell differentiation GO terms (Figure S4A,B). Importantly, 2C embryo‐specific transcripts such as *Zscan4c*, *Duxbl1*, *Usp17lc*, *Tcstv1*, *Gata2* and *Gadd45a* were upregulated in *Nelfa* + *Bcl2* OE ESCs (Figure 4A,B), whereas the downregulated genes included pluripotency genes such as *Lin28b*, *Lefty2*, *Bmp4* and *Lin28a* (Figure 4A). Meanwhile, the expression levels of some pluripotency genes, such as *Oct4* (*Pou5f2*), *Klf4*, *Lifr* and *Fgf4*, did not change (Figure 4B). Of the 898 genes previously reported as being pluripotency‐specific,^13^ 805 were found to be expressed in *Nelfa* OE ESCs, while only 63 of these were differentially expressed (Figure 4C). These data indicated that the expression levels of pluripotency genes remain mostly unchanged in *Nelfa* + *Bcl2* OE ESCs and the cells retain a pluripotency state.

In contrast, 27.9% of *Nelfa*‐induced genes^13^ and 9.4% of 2C embryo‐specific genes^13^ were upregulated in *Nelfa* + *Bcl2* OE ESCs, including *Zscan4f, Zscan4c Zscan4d*, *Usp17lb*, *Tcstv1* and *Usp17lc* (Figure 4D). To further investigate the 2C‐related features of *Nelfa* + *Bcl2* OE ESCs, we compared the genes upregulated in *Nelfa* + *Bcl2* OE ESCs with those induced by *Zscan4*,^17^ *Dux*,^29^ *Chaf1a* depletion^29^ and *Nelfa*.^13^ A Venn diagram showed that numerous 2C genes overlapped among the five groups, such as *Nelfa*, *Tdpoz4*, *Zscan4d*, *Zscan4c*, *Usp17la*, *Zscan4e*, *Tcstv3*, *Usp17lc*, *Zscan4f*, *Usp17le* and *Zfp352* (Figure 4E). However, only relatively few 2C genes overlapped between the five above‐mentioned groups and *Nelfa* OE ESCs, which further confirmed that the overexpression of *Nelfa* alone in naïve ESCs can only partially activate the 2C‐like state in these cells (Figure S4C,D).

Two cell‐like cells show unique epigenetic features, including flexible chromatin accessibility, reduced DNA methylation and ‘active’ histone modification.^7^, ^9^, ^14^ Therefore, we wondered whether *Nelfa* + *Bcl2* OE ESCs exhibit some of the epigenetic modification characteristics associated with 2C‐like cells. Transcriptional analysis showed that the expression of most histone deacetylation‐related genes was significantly reduced in *Nelfa* + *Bcl2* OE ESCs compared with that in WT ESCs (Figure 4F). As expected, H3K27 acetylation (H3K27ac), the ‘active’ histone mark, was significantly increased in *Nelfa* + *Bcl2* OE ESCs compared with that observed in WT ESCs (Figure 4G). Additionally, genes that are negatively correlated with 2C‐like cells were significantly downregulated in *Nelfa* + *Bcl2* OE ESCs (Figure S4E), including those associated with chromatin condensation,^17^, ^45^, ^46^ DNA methylation^14^, ^15^ and DNA replication fork speed.^19^, ^20^ Moreover, consistent with previous reports, the expression of genes associated with the exit from naïve pluripotency^13^, ^42^ was significantly higher in *Nelfa* + *Bcl2* OE ESCs than in WT ESCs (Figure S4E). These results demonstrated that the transcriptional features of *Nelfa* + *Bcl2* OE ESCs highly resemble those of the 2C‐like state.

### 3.5 Overexpression of *Bcl2* alone induces a 2C‐like state in ESCs

**FIGURE 5:**
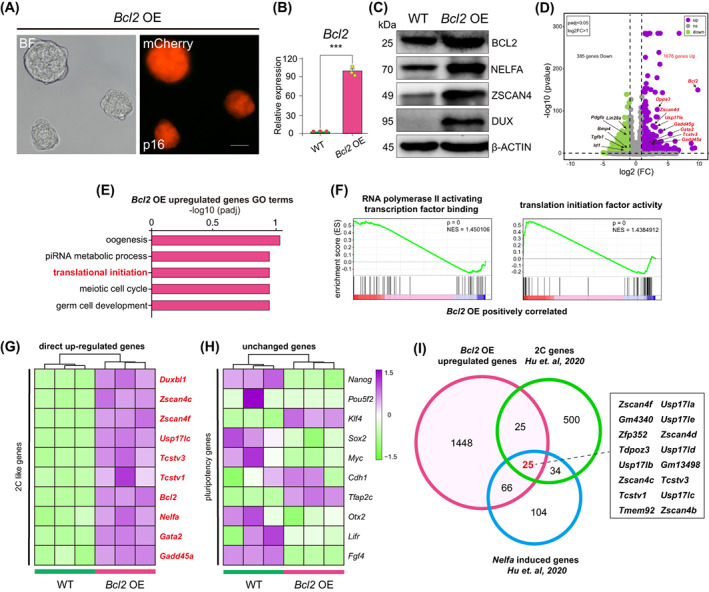
The overexpression of *Bcl2* alone drives the 2C‐like state in embryonic stem cells (ESCs). (A) Brightfield and fluorescence images showing *Bcl2* overexpressing (OE) ESCs carrying mCherry. Scale bars, 100 μm. (B) The relative expression level of *Bcl2* in *Bcl2* OE ESCs and wild‐type (WT) ESCs as determined by RT‐qPCR. Error bars represent means ± SD (*n* = 3). The *p*‐values were calculated using two‐tailed Student's *t*‐tests, *p* < 0.05; *n* = 3 biological replicates. (C) Western blot of BCL2, NELFA, ZSCAN4 and DUX in WT ESCs and *Bcl2* OE ESCs. β‐Actin was used as a loading control. (D) Volcano plot showing the differentially expressed genes (DEGs; green: downregulated, purple: upregulated) between *Bcl2* OE ESCs and WT ESCs; some DEGs are listed. (E) Gene ontology (GO) analysis of indicated biological processes of *Bcl2* OE upregulated genes in *Bcl2* OE ESCs compared with WT ESCs were significantly enriched. (F) The genes upregulated in *Bcl2* OE ESCs relative to WT ESCs were highly associated with translational initiation and RNA polymerase II activating transcription factor binding process as determined by gene set enrichment analysis (GSEA). Normalised enrichment scores (NES) and nominal *p*‐values are shown. (G,H) A heatmap showing the expression levels of 2C‐like genes (G) and pluripotency genes (H) in *Bcl2* OE ESCs and WT ESCs. (I) A Venn diagram showing that 25 genes that were upregulated in *Bcl2* OE ESCs were previously identified as 2C‐related genes.

Next, we asked whether the overexpression of *Bcl2* alone is sufficient to induce a 2C‐like state. To assess this possibility, we generated a Dox‐inducible *Bcl2*‐mCherry (*Bcl2* OE) ESC line (Figure 5A). *Bcl2* OE ESCs expressed high levels of *Bcl2* mRNA and protein (Figure 5B,C). Immunostaining also indicated that the levels of key pluripotency‐related proteins, such as OCT4, SOX2 and NANOG, were unaffected by *Bcl2* overexpression (Figure S5A). Moreover, *Bcl2* OE ESCs retained the capacity for spontaneous differentiation in vitro (Figure S5B).

To better characterise the *Bcl2* OE ESCs, we performed RNA‐seq analysis. We observed robust upregulation of key 2C genes (Figure 5D), and the upregulated genes were found to be highly associated with translational initiation and RNA polymerase II activating transcription factor binding process (Figure 5E,F), consistent with molecular events that occur in early embryos for reprogramming towards totipotency.^16^, ^47^, ^48^, ^49^ In contrast, the genes upregulated in WT ESCs were mainly associated with developmental processes, including bone morphogenesis, pattern specification process and embryonic organ development (Figure S5C). Although key 2C genes and proteins were markedly upregulated in *Bcl2* OE ESCs (Figure 5C,D,G, and Figure S5D), no differences in the expression levels of most pluripotency genes were observed (Figure 5H). Of the 898 pluripotency‐specific genes previously reported,^13^ 806 were also expressed in *Bcl2* OE ESCs, and only 34 were differentially expressed in *Bcl2* OE ESCs relative to WT ESCs (Figure S5E).

The genes upregulated in *Bcl2* OE ESCs overlap with those reported to be significantly upregulated in other cell models of the 2C‐like state (2C embryo‐specific genes,^13^ *Nelfa*‐induced genes,^13^ *Dux*‐induced genes,^29^ *Zscan4*‐positive genes^17^ and genes induced by *Chaf1a* depletion^29^). The 2C genes showing the greatest overlap included *Zscan4c*, *Zscan4e*, *Tdpoz3*, *Tcstv3*, *Usp17lc* and *Zfp352* (Figure 5I and Figure S5F). Meanwhile, the genes that were significantly downregulated in *Bcl2* OE ESCs (Figure S5G) included negative regulators of 2C‐like cells, such as genes associated with chromatin condensation,^13^, ^46^ DNA methylation,^14^, ^15^ and DNA replication fork speed.^19^, ^20^ The expression of genes involved in the exit from naïve pluripotency^13^, ^42^ was significantly higher in *Bcl2* OE ESCs than in WT ESCs (Figure S5G).^13^, ^42^ These data demonstrated that the overexpression of *Bcl2* alone in ESCs can induce a 2C‐like state.

**FIGURE 6:**
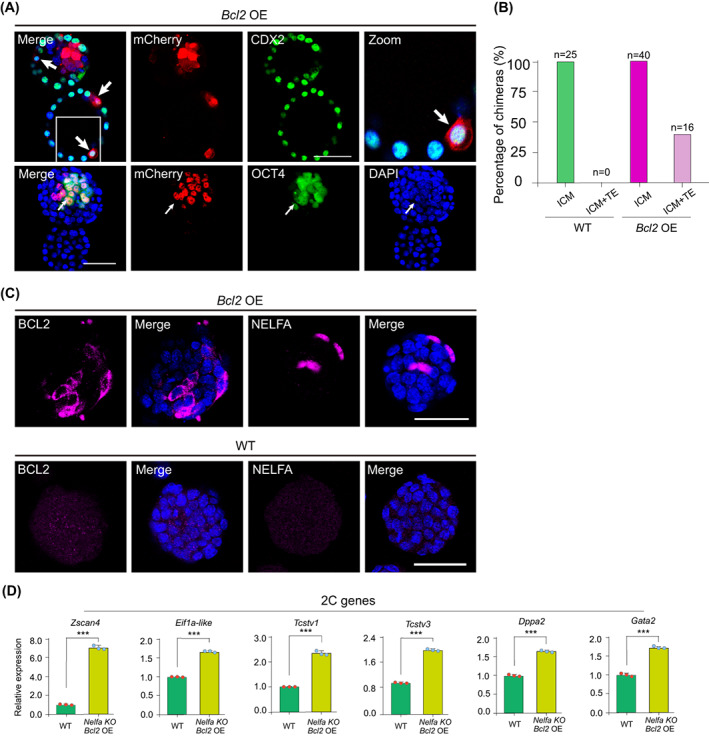
*Bcl2* overexpression activates endogenous NELFA expression. (A) Representative images of chimeric blastocyst immunostaining. Eight‐cell stage embryos were injected with mCherry‐labelled *Bcl2* OE ESCs and then cultured for 48 h in vitro. mCherry‐labelled *Bcl2* OE ESCs contributed to the OCT4‐positive inner cell mass (ICM) and CDX2‐positive trophectoderm (TE). Scale bars, 50 μm. (B) Summary of E4.5 chimera assays using *Bcl2* OE ESC injection (*n* = 3 independent experiments). (C) Immunofluorescence staining for BCL2 and NELFA in *Bcl2* OE ESCs (top) and wild‐type (WT) ESCs (bottom). Cell nuclei were stained with 4′,6‐diamidino‐2‐phenylindole (DAPI). Scale bar, 50 μm. (D) RT‐qPCR analysis of 2C genes (*Zscan4*, *Eif1a‐lik*e, *Tcstv1*, *Tcstv3*, *Dppa2* and *Gata2*) in *Nelfa* KO ESCs overexpressing *Bcl2*. Error bars represent means ± SD (*n* = 3). The *p*‐values were calculated using two‐tailed Student's *t*‐tests, *p* < 0.05; *n* = 3 biological replicates.

The key feature of 2C‐like cells is that they can contribute to both embryonic and extraembryonic lineages in chimeras. To assess the developmental potential of *Bcl2* OE ESCs in vivo, we injected mCherry‐labelled *Bcl2* OE ESCs into 8‐cell stage embryos and generated chimeric blastocysts. We found that *Bcl2* OE ESCs contributed to both the ICM and the TE (Figure 6A,B). These observations imply that ESCs that overexpress *Bcl2* exhibit expanded cell fate potential, which is the hallmark of 2C‐like cells.

### 3.6 *Bcl2* overexpression activates a 2C‐like state in a NELFA‐independent manner

Next, we investigated whether a regulatory relationship exists between *Nelfa* and *Bcl2*. Interestingly, when *Bcl2* was overexpressed in WT ESCs, NELFA expression was upregulated at the protein level, as determined by both western blotting and immunofluorescence staining (Figures 5C and 6C). Accordingly, we next assessed whether the BCL2‐mediated induction of a 2C‐like state was NELFA‐dependent. We overexpressed *Bcl2* in *Nelfa* KO ESCs and noted that the expression of 2C genes, including *Zscan4*, *Eif1a‐like*, *Tcstv1*, *Tcstv3*, *Dppa2* and *Gata2*, was significantly upregulated in the ESCs (Figure 6D). These findings indicated that the overexpression of *Bcl2* can robustly induce the 2C‐like state in *Nelfa* KO ESCs, indicating that the role of BCL2 in the induction of a 2C‐like state is independent of NELFA.

### 3.7 *Nelfa* promotes the efficiency of somatic cell reprogramming

**FIGURE 7:**
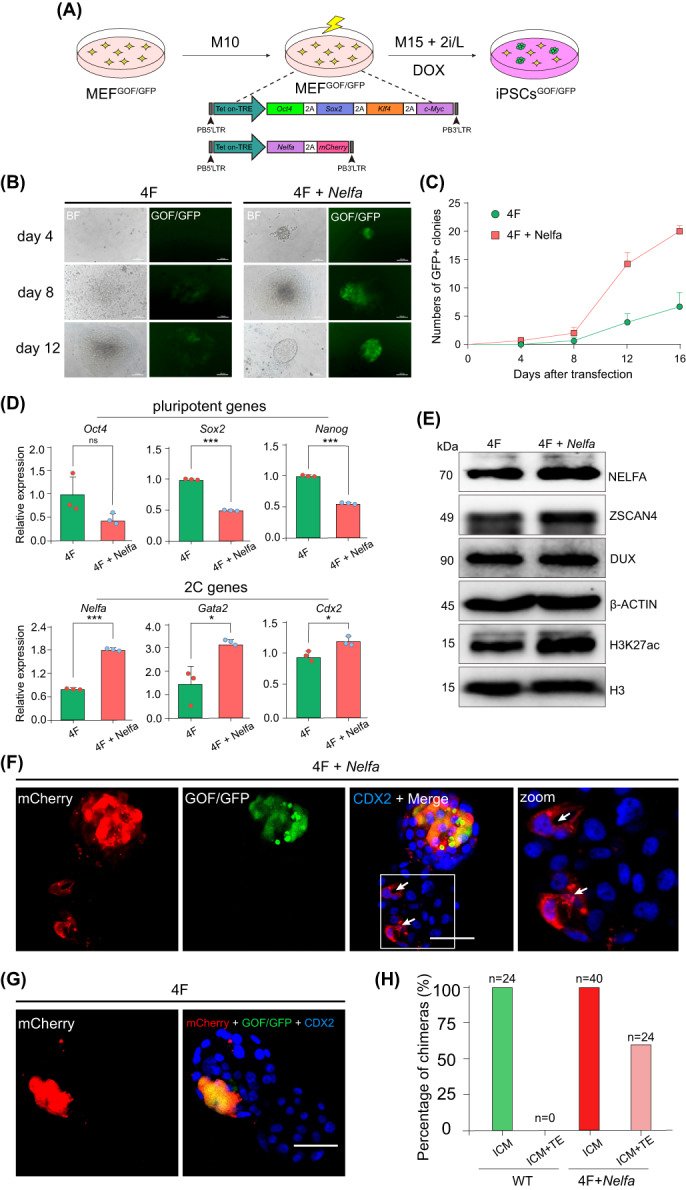
*Nelfa* enhances the efficiency of somatic cell reprogramming. (A) Scheme of the piggyBac‐based vectors used for the generation of induced pluripotent stem cells (iPSCs) from MEF^GOF/GFP^. (B) Representative brightfield and fluorescence images showing the changes in the 4F‐ transfected group (left) and 4F + *Nelfa* transfected group (right) at the indicated time points during iPSC generation. Scale bar, 100 μm. (C) The numbers of GFP^+^ clones of iPSCs increased after the transfection of 4F and 4F + *Nelfa*. Error bars indicate means ± SD (*n* = 3). *P*‐values were calculated using two‐tailed Student's *t*‐tests, *p* < 0.05; *n* = 3 biological replicates. (D) The relative expression levels of pluripotency genes (*Oct4*, *Sox2* and *Nanog*) and 2C genes (*Nelfa*, *Gata2* and *Cdx2*) in 4F + *Nelfa* iPSCs and 4F iPSCs as determined by RT‐qPCR. Error bars represent means ± SD (*n* = 3). The *p*‐values were calculated using two‐tailed Student's *t*‐tests, *p* < 0.05; *n* = 3 biological replicates. (E) Western blot of NELFA, ZSCAN4, DUX and H3K27ac in 4F + *Nelfa* iPSCs and 4F iPSCs. β‐Actin and H3 served as loading controls. (F,G) Representative images showing immunostaining of chimeric blastocysts injected with mCherry‐labelled 4F + *Nelfa* iPSCs and 4F iPSCs. 4F + *Nelfa* iPSCs contributed to both the inner cell mass (ICM) and trophectoderm (TE) (F) in chimeric blastocysts. However, 4F iPSCs contributed exclusively to the ICM (G). (H) A summary of E4.5 chimera assays using 4F + *Nelfa* iPSC and 4F iPSC injection (*n* = 3 independent experiments).

It has been reported that 2C genes such as *Zscan4* and *Dux*,^24^, ^25^, ^26^ if included in the reprogramming cocktail along with the four core reprogramming factors *Oct4*, *Sox2*, *Klf4* and *c‐Myc* (OKSM),^50^ significantly enhance somatic cell reprogramming efficiency. We postulated that the upregulation of *Nelfa* in conjunction with Yamanaka's four factors may augment the efficacy of iPSC generation. To test this possibility, we transfected a piggyBac Tet on‐TRE vector containing Dox‐inducible OKSM (4F) or 4F plus a piggyBac Tet on‐TRE vector harbouring Dox‐inducible *Nelfa* (4F + *Nelfa*) into MEF^GOF/GFP^. After transfection, the cells were cultured in 2i/L medium supplemented with FBS and Dox (Figure 7A). We observed the cells under a fluorescence microscope daily and found that ESC‐like GOF/GFP‐positive colonies appeared as early as 4 days after the transfection of 4F + *Nelfa* (Figure 7B), indicating that these cells expressed high levels of endogenous *Oct4*. In the 4F group, GOF/GFP‐positive colonies started appearing on day 8 after transfection (Figure 7B). Importantly, there were significantly more GOF/GFP‐positive colonies in the 4F + *Nelfa* group than in the 4F group, counted by different day after transfection (Figure 7C). These results indicated that the co‐expression of *Nelfa* with Yamanaka's four factors can enhance the speed and efficiency of MEF reprogramming.

Next, we investigated whether the overexpression of *Nelfa* in iPSCs also induces a 2C‐like state in these cells. As expected, although the expression levels of *Oct4*, *Sox2* and *Nanog* were slightly decreased in 4F + *Nelfa* iPSCs, some 2C genes (*Nelfa*, *Gata2* and *Cdx2*) were upregulated compared with those of 4F iPSCs (Figure 7D). Notably, the protein levels of 2C‐associated ZSCAN4, DUX and H3K27ac were higher in 4F + *Nelfa* iPSCs than in 4F iPSCs (Figure 7E). Next, we microinjected mCherry‐labelled 4F + *Nelfa* iPSCs and mCherry‐labelled 4F iPSCs into 8‐cell stage embryos and analysed the resulting chimeric blastocysts. We found that 4F + *Nelfa* iPSCs colonized both the ICM and the TE (Figure 7F,H), whereas 4F iPSCs contributed only to the ICM of chimeric blastocysts (Figure 7G). These findings demonstrated that overexpressing *Nelfa* in iPSCs also confers dual cell fate potential in chimeric embryos.

## 4 DISCUSSION

In this study, we demonstrated that *Bcl2* overexpression drives 2C‐like state conversion in ESCs in naïve conditions, and provided insights into how *Nelfa* and *Bcl2* mediate the conversion of ESCs from a pluripotent to a 2C‐like state at the transcriptional level. Our investigations confirmed that although *Nelfa* can partially activate the 2C‐like state in ESCs under naïve culture conditions, *Nelfa* depletion could not block this activation. Moreover, our results indicated that *Bcl2* can induce endogenous NELFA expression and the 2C‐like state in ESCs even in *Nelfa* KO ESCs, indicating that the BCL2‐mediated conversion of the 2C‐like state is NELFA‐independent. Importantly, we also found that *Nelfa* significantly enhances somatic cell reprogramming efficiency. This finding provides insight into the establishment and regulation of the totipotent state in mouse embryonic stem cells.

Most studies investigating the conversion from a pluripotent to a 2C‐like state have employed serum‐containing conditions.^7^, ^13^ However, it has been reported that naïve culture conditions could not induce this conversion.^13^ Here, we showed that the overexpression of *Nelfa* in ESCs can partially initiate the 2C‐like state in ESCs under naïve culture conditions. Nevertheless, unlike with serum‐containing media, it is challenging to fully achieve a 2C‐like state by overexpressing *Nelfa* solely under naïve culture conditions. These observations suggest that serum contains the key components necessary for 2C‐like state activation; however, the composition of serum is complex, and it is challenging to identify which factors in serum play a key role in this process. Importantly, we found that although the overexpression of *Nelfa* can initiate the 2C‐like state, the depletion of *Nelfa* did not affect ESC pluripotency and did not completely block the activation of the 2C‐like state in ESCs, indicating that *Nelfa* is dispensable for this process. Thus, the precise role of *Nelfa* in the conversion from a pluripotent to a 2C‐like state remains to be fully elucidated. Embryos with maternal zygotic *Nelfa* depletion will help to clarify the role of *Nelfa* in the 2C‐like state as well as in ZGA.

Two cell‐like cells are unstable in culture and no suitable culture condition is currently available for the maintenance and self‐renewal of these cells. There is indirect evidence linking increased DNA damage and cell death with a pluripotent‐to‐totipotent state conversion.^18^, ^20^ DNA damage induced by ultraviolet light irradiation, zeocin, doxorubicin and hydroxyurea‐aphidicolin treatment was reported to promote the transition to a 2C‐like state in a p53‐dependent manner.^20^ In contrast, the 2C‐like state in ESCs in vitro culture was partially abrogated by the loss of p53. Double‐stranded DNA breaks directly or indirectly promote the *Dux*‐ and *Zscan4*‐mediated induction of the 2C‐like state in ESCs, and 2C‐like cells exhibit high levels of phosphorylated H2A.X (γH2A.X).^19^, ^51^ Here, we demonstrated that the overexpression of the anti‐apoptotic gene *Bcl2* alone or in combination with *Nelfa* can robustly promote the expression of 2C‐like transcripts in ESCs as well as endow the cells with dual cell fate (ICM and TE) potential. Meanwhile, we also found that the co‐overexpression of *Nelfa* and *Bcl2* slightly decreased the level of γH2A.X compared with *Nelfa* overexpression alone, indicating the *Bcl2* overexpression may promote the 2C‐like state conversion via other mechanisms besides DNA damage, which would open up a new path towards capturing 2C‐like cells from ESCs. The precise relationship among DNA damage, cell death and anti‐apoptotic genes related to a 2C‐like state requires further exploration.

Our results further showed that the co‐expression of *Nelfa* with Yamanaka's four factors can enhance the speed and efficiency of the reprogramming of MEFs into iPSCs, and *Nelfa*‐overexpressing iPSCs also show a 2C‐like state, consistent with previous reports that strategies used for the induction of a 2C‐like state can improve the efficiency of SCNT or somatic cell reprogramming.^24^, ^25^, ^26^ Thus, 2C‐like cells may represent an ideal cell model for studying certain aspects of SCNT.^52^ Our finding of the combined effect of *Nelfa* and *Bcl2* in mediating the induction of the 2C‐like state provides a valuable in vitro model for dissecting common regulators or pathways involved in the control of totipotency.

## AUTHOR CONTRIBUTIONS

Baojiang Wu, Siqin Bao, Xihe Li and Jun Liu conceived and designed the project. Yanqiu Wang, Baojiang Wu and Xinhua Wei performed most experiment and analysed data. Jingcheng Zhang, Guifang Cao and Yong Zhang provided technical assistance. Baojiang Wu, Yanqiu Wang, Siqin Bao, Xihe Li and Jun Liu wrote the manuscript with contributions from all authors.

## CONFLICT OF INTEREST STATEMENT

The authors declare that they have no conflict of interest.

## Supporting information


**Data S1.** Supporting Information.

## Data Availability

The RNA‐seq datasets are available at the NCBI Sequence Read Archive (SRA) under the ID PRJNA940854. Original blots and microscopy images are available from the lead contact upon request.
