# Supplementary material for: NELFA and BCL2 induce the 2C‐like state in mouse embryonic stem cells in a chemically defined medium
Source: Cell Prolif. 2023 Aug 17;57(2):e13534. doi: 10.1111/cpr.13534 (PMC10849787; doi:10.1111/cpr.13534)

Supporting information

Figure S1. *Nelfa* overexpression induces the 2C-like state of ESCs. Related to Figure 1.

A, Scheme for the PiggyBac-based Tet-on-TRE-*Nelfa-mCherry* vectors to induce the *Nelfa* OE ESCs.

B, Relative expression levels of pluripotency genes (*Oct4*, *Sox2*, *Nanog*) in *Nelfa* OE ESCs versus WT ESCs, as determined by RT-qPCR. Error bars are mean ± SD (n = 3). *P* values were calculated by two tailed Student's *t*-test, $p < 0.05$. n=3 biological replicates.

C, Representative immunostaining for OCT4, SOX2, NANOG in WT ESCs and *Nelfa* OE ESCs. DAPI staining delineates cell nuclei. Scale bar, 50 μm.

D, Heat map showing expression levels of pluripotency genes and *Nelfa* induced genes in WT ESCs and *Nelfa* OE ESCs. Right: Venn diagram indicated that *Nelfa* induced genes and pluripotency genes in *Nelfa* OE ESCs compared with previous reported.

E, Bright field images showing *Nelfa* OE ESCs before and after differentiation (left). Scale bar, 100 μm. Relative expression levels of pluripotency genes in *Nelfa* OE ESCs versus WT ESCs after spontaneously differentiation, as determined by RT-qPCR. Error bars are mean ± SD (n = 3). *P* values were calculated by two tailed Student's *t*-test, $p < 0.05$. n=3 biological replicates.

Figure S2. Nelfa knockout could not block the 2C-like state of ESCs. Related to Figure 2.

A, Scheme for establishing a *Nelfa* KO ESCs line by CRISPR -Cas9 co-transfected with EGFP.

B, EGFP-labelled *Nelfa* KO ESCs contributed to the epiblast of gastrula stage embryos. Scale bars, 100 μm.

C, The summary of epiblast (E6.5) contribution derived from *Nelfa* KO ESCs (n = 3 independent experiments).

Figure S3. Nelfa and Bcl2 overexpression activate the 2C-like state of ESCs.

Related to Figure 3.

A, Immunofluorescence staining for OCT4, SOX2 and NANOG in WT ESCs and *Nelfa* + *Bcl2* OE ESCs. Cell nuclei are stained with DAPI (4′,6-diamidino-2-phenylindole). Scale bar, 50 μm.

B, Representative images showing immunostaining of chimeric blastocysts injected with WT ESCs at 8-cell stage embryos, which were then cultured 48 h *in vitro*, and showed that injected cells contributed exclusively to the ICM. Scale bars, 50 μm.

C, mCherry-labelled *Nelfa* + *Bcl2* OE ESCs were injected into 8-cell stage embryos, which were then cultured 48 h *in vitro*. *Nelfa* + *Bcl2* OE ESCs were able to contribute to OCT4 positive ICM. Scale bars, 50 μm.

D, Bright field images showing *Nelfa* + *Bcl2* OE ESCs before and after differentiation (left). Scale bar, 100 μm. Relative expression levels of pluripotency genes in *Nelfa* + *Bcl2* OE ESCs versus WT ESCs after spontaneously differentiation, as determined by RT-qPCR. Error bars are mean ± SD (n = 3). *P* values were calculated by two tailed Student's *t*-test, $p < 0.05$. n=3 biological replicates.

Figure S4. Overexpression of Nelfa and Bcl2 induce the 2C transcriptional program in ESCs. Related to Figure 4.

A and **B**, Gene Ontology (GO) analysis of indicated biological processes of downregulated genes (log2FC > 1, padj < 0.05) (**A**) and the upregulated genes (**B**) in *Nelfa* + *Bcl2* OE ESCs compared with WT ESCs were significantly enriched.

C, Venn diagram showed that only 5 genes were overlapped in *Nelfa* OE ESCs upregulated genes compared with previously published *Zscan4* positive upregulated, *Dux* induced, *Chaf1a* depletion and *Nelfa* induced genes.

D, Venn diagram showed that 4 genes were overlapped in *Nelfa* + *Bcl2* OE ESCs upregulated genes compared with *Nelfa* OE ESCs upregulated genes, *Zscan4* positive upregulated, *Dux* induced, *Chaf1a* depletion and *Nelfa* induced genes.

E, The heatmap showing expression levels of 2C-like cells negative regulation related chromatin condensation, DNA methylation and DNA replication fork speed associated genes in *Nelfa* + *Bcl2* OE ESCs compared with WT ESCs.

Figure S5. Overexpression of Bcl2 alone can derives 2C-like state of ESCs. Related to Figure 5.

A, Immunofluorescence staining for the pluripotent proteins (OCT4, SOX2 and NANOG) in WT ESCs and *Bcl2* OE ESCs. Cell nuclei are stained with DAPI (4′,6-diamidino-2-phenylindole). Scale bar, 50 μm.

B, Relative expression of mesoderm, endoderm, and ectoderm genes measured by RT-qPCR, following spontaneously differentiation of WT ESCs and *Bcl2* OE ESCs *in vitro*. Error bars are mean ± SD (n = 3). *P* values were calculated by two tailed Student's *t*-test, $p < 0.05$. n=3 biological replicates.

C, Gene Ontology (GO) analysis of indicated biological processes of WT ESCs upregulated genes were significantly enriched.

D, Immunofluorescence staining for ZSCAN4 and MERVL in WT ESCs (left) and *Bcl2* OE ESCs (right). Cell nuclei are stained with DAPI (4′,6-diamidino-2-phenylindole). Scale bar, 50 μm.

E, Venn diagrams showing that 806 genes were detected in *Bcl2* OE ESCs compared with 898 pluripotency specific genes published previously.

F, Venn diagram showed that 45 genes were overlapped in *Bcl2* OE ESCs upregulated genes, and previously published *Zscan4* positive upregulated, *Dux* induced, *Chaf1a* depletion and *Nelfa* induced genes, such as *Zscan4c*, *Tdpoz3*, *Zscan4d* and *Usp17le.*

G, The heatmap showing expression levels of 2C-like cells negative regulation related chromatin condensation, DNA methylation and DNA replication fork speed associated and exit from naïve pluripotency related genes in *Nelfa* + *Bcl2* OE ESCs compared with WT ESCs.

Figure S1

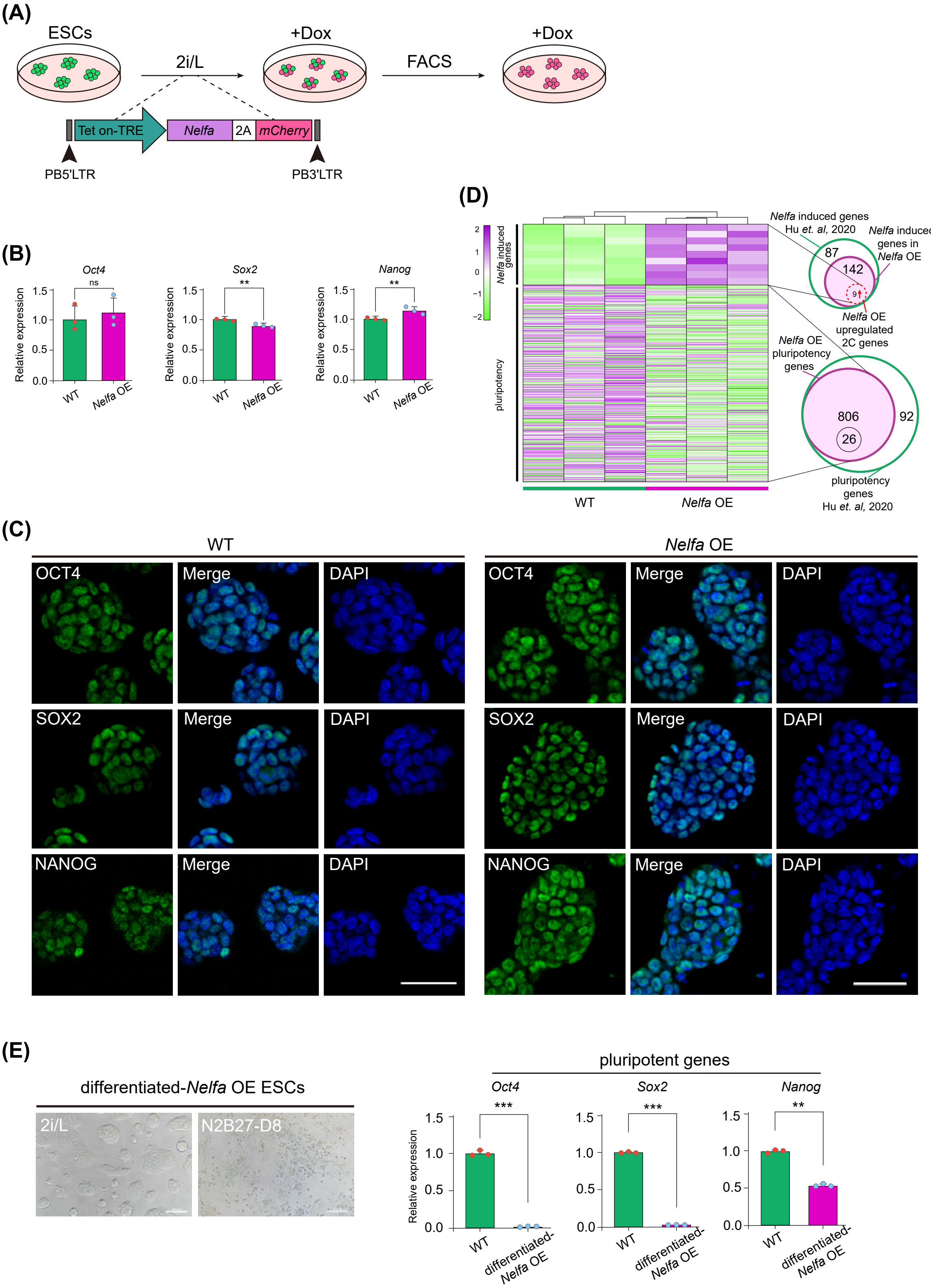

(A)
ESCs
2i/L
+Dox
FACS
+Dox
Tet on-TRE
Nelfa
2A
mCherry
PB5'LTR
PB3'LTR
(B)
Oct4
Sox2
Nanog
ns
**
**
Relative expression
WT
Nelfa OE
(C)
WT
Nelfa OE
OCT4
Merge
DAPI
SOX2
NANOG
(D)
Nelfa induced genes
pluripotency
Nelfa induced genes Hu et. al, 2020
Nelfa induced genes in Nelfa OE
87
142
9
Nelfa OE upregulated 2C genes
Nelfa OE pluripotency genes
806
26
92
pluripotency genes Hu et. al, 2020
WT
Nelfa OE
(E)
differentiated-Nelfa OE ESCs
2i/L
N2B27-D8
pluripotent genes
Oct4
Sox2
Nanog
***
***
**
Relative expression
WT
differentiated-Nelfa OE

Figure S2

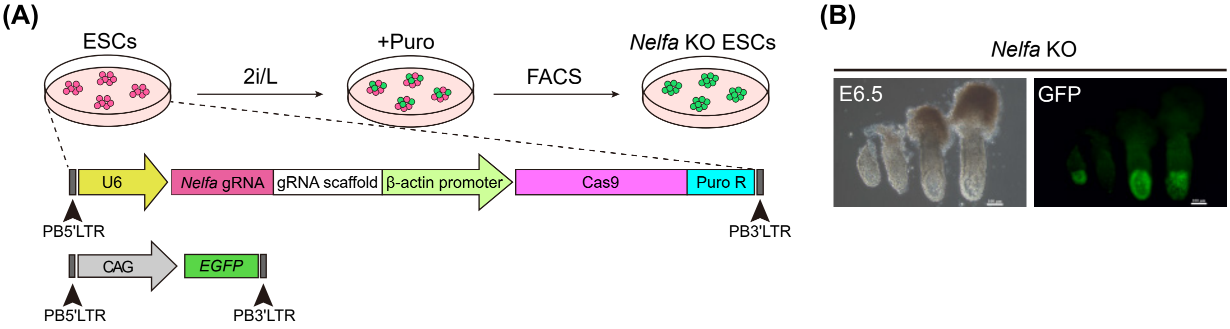

(C)

Cell lines	Stage of embryos	No. of injected cells	No. of collected	epiblast (E6.5) contribution (%)
Nelfa KO, p14	8-cell	8-12	5	4 (80%)

Figure S3

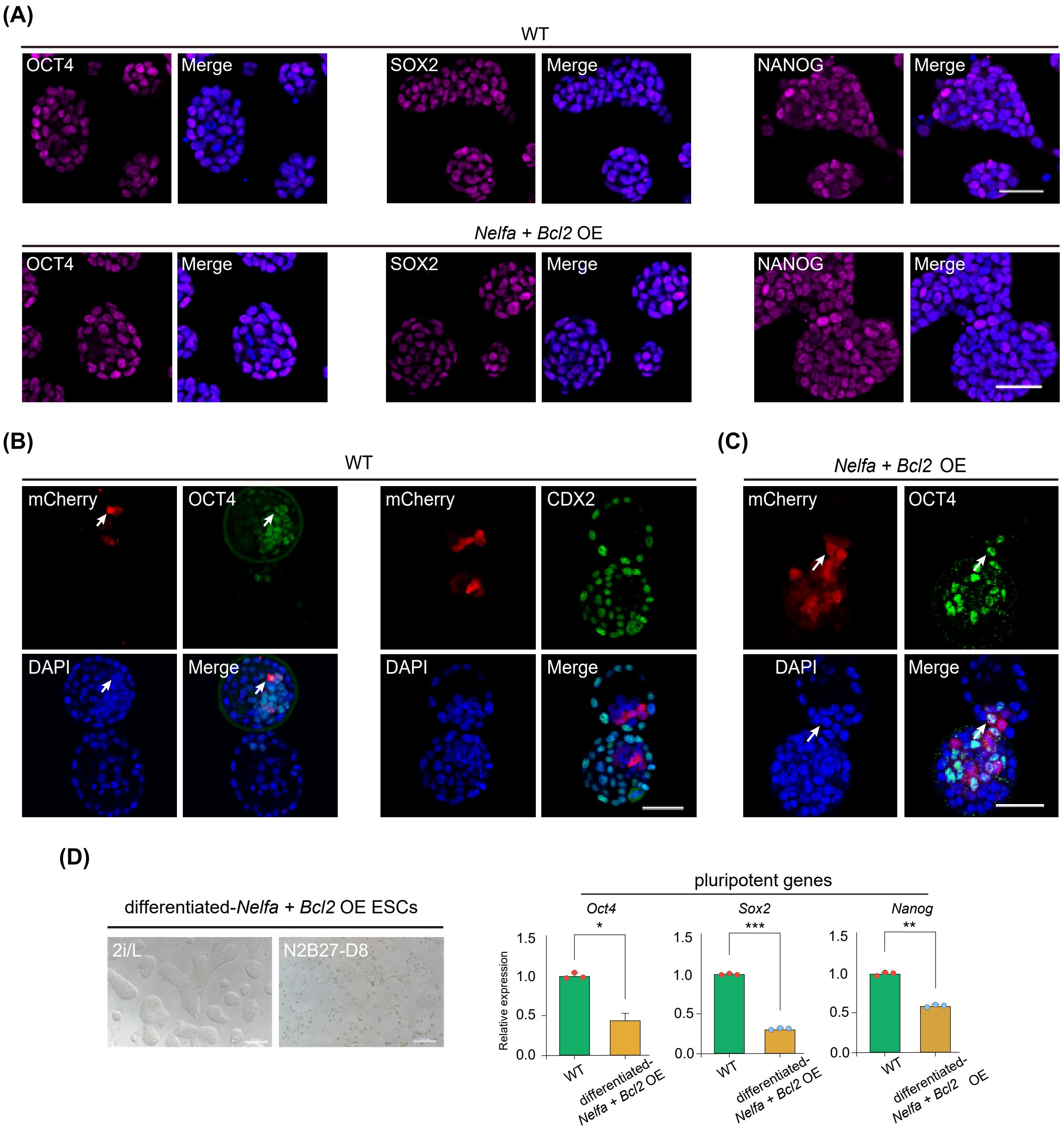

(A)
WT
OCT4
Merge
SOX2
Merge
NANOG
Merge
Nelfa + Bcl2 OE
OCT4
Merge
SOX2
Merge
NANOG
Merge
(B)
WT
mCherry
OCT4
mCherry
CDX2
DAPI
Merge
DAPI
Merge
(C)
Nelfa + Bcl2 OE
mCherry
OCT4
DAPI
Merge
(D)
differentiated-Nelfa + Bcl2 OE ESCs
2i/L
N2B27-D8
pluripotent genes
Oct4
Sox2
Nanog
*
***
**
Relative expression
1.5
1.0
0.5
0.0
WT
differentiated-Nelfa + Bcl2 OE

Figure S4

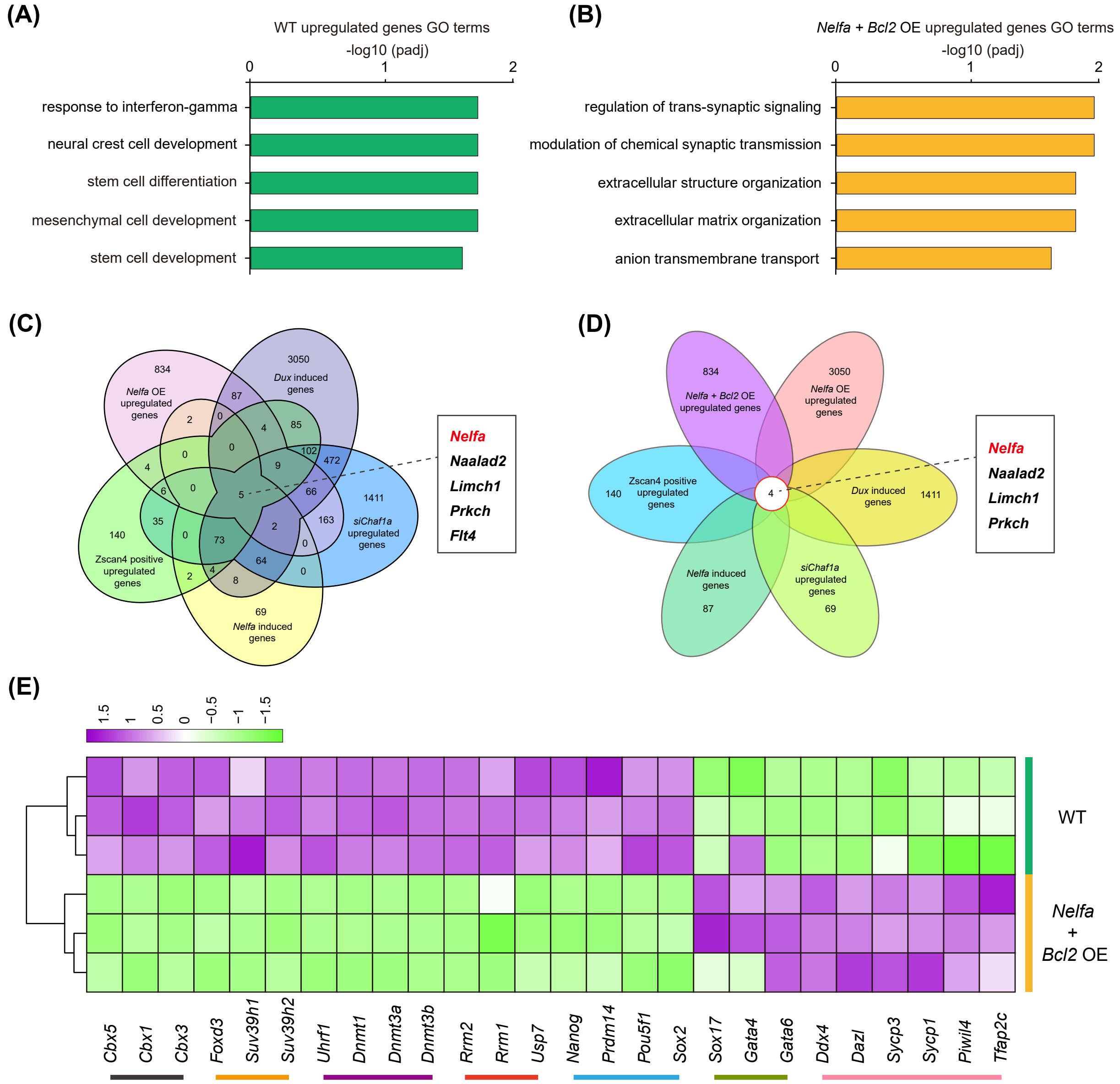

(A)
WT upregulated genes GO terms
-log10 (padj)
response to interferon-gamma
neural crest cell development
stem cell differentiation
mesenchymal cell development
stem cell development
(B)
Nelfa + Bcl2 OE upregulated genes GO terms
-log10 (padj)
regulation of trans-synaptic signaling
modulation of chemical synaptic transmission
extracellular structure organization
extracellular matrix organization
anion transmembrane transport
(C)
Nelfa OE upregulated genes
Dux induced genes
siChaf1a upregulated genes
Nelfa induced genes
Zscan4 positive upregulated genes
Nelfa
Naalad2
Limch1
Prkch
Flt4
(D)
Nelfa + Bcl2 OE upregulated genes
Nelfa OE upregulated genes
Dux induced genes
siChaf1a upregulated genes
Nelfa induced genes
Zscan4 positive upregulated genes
Nelfa
Naalad2
Limch1
Prkch
(E)
WT
Nelfa + Bcl2 OE
Cbx5 Cbx1 Cbx3 Foxd3 Suv39h1 Suv39h2 Uhrf1 Dnmt1 Dnmt3a Dnmt3b Rrm2 Rrm1 Usp7 Nanog Prdm14 Pou5f1 Sox2 Sox17 Gata4 Gata6 Ddx4 Dazl Sycp3 Sycp1 Piwil4 Tfap2c

Figure S5

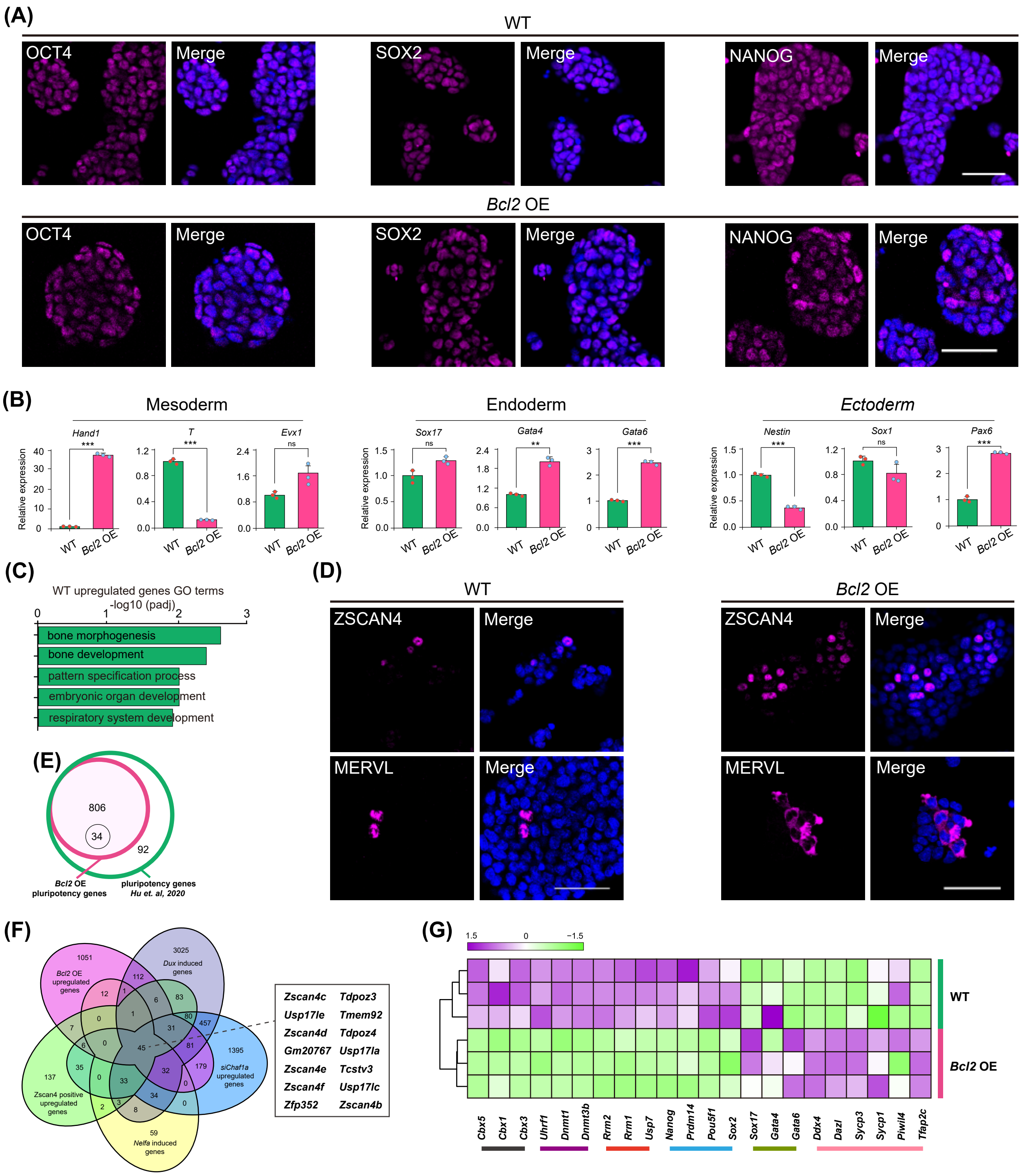

(A)
WT
OCT4
Merge
SOX2
Merge
NANOG
Merge
Bcl2 OE
OCT4
Merge
SOX2
Merge
NANOG
Merge
(B)
Mesoderm
Endoderm
Ectoderm
Hand1
T
Evx1
Sox17
Gata4
Gata6
Nestin
Sox1
Pax6
Relative expression
WT
Bcl2 OE
(C)
WT upregulated genes GO terms
-log10 (padj)
bone morphogenesis
bone development
pattern specification process
embryonic organ development
respiratory system development
(D)
WT
Bcl2 OE
ZSCAN4
Merge
MERVL
Merge
(E)
806
34
92
Bcl2 OE pluripotency genes
pluripotency genes Hu et. al, 2020
(F)
Bcl2 OE upregulated genes
Dux induced genes
siChaf1a upregulated genes
Nelfa induced genes
Zscan4 positive upregulated genes
Zscan4c
Tdpoz3
Usp17le
Tmem92
Zscan4d
Tdpoz4
Gm20767
Usp17la
Zscan4e
Tcstv3
Zscan4f
Usp17lc
Zfp352
Zscan4b
(G)
WT
Bcl2 OE
Cbx5
Cbx1
Cbx3
Uhrf1
Dnmt1
Dnmt3b
Rrm2
Rrm1
Usp7
Nanog
Prdm14
Pou5f1
Sox2
Sox17
Gata4
Gata6
Ddx4
Dazl
Sycp3
Sycp1
Piwil4
Tfap2c

Table S1. gRNA sequences

Name	Sequence (5' to 3')
Nelfa-g1-F	CACCGATGTTATCGATGACCGCGG
Nelfa-g1-R	AAACCCGCGGTCATCGATAACATC
Nelfa-g2-F	CACCGTGGCGCCCACAGCTCGTCCG
Nelfa-g2-R	AAACCGGACGAGCTGTGGGCGCCAC
Nelfa-g3-F	CACCGTGAGCAGGGACGCGATGCT
Nelfa-g3-R	AAACAGCATCGCGTCCCTGCTCAC

Table S2. Primary antibody for immunostaining

Name	Dilution ratio
anti-OCT4 (BD Biosciences, 611203)	1:200 mouse
anti-SOX2 (R&D Systems, AF2018)	1:200 goat
anti-NANOG (eBioscience, 14-5761)	1:500 rat
anti-CDX2 (BioGenex, AM392)	1:200 mouse
anti-BCL2 (Elabscience, E-AB-60012)	1:100 rabbit
anti-NELFA (Bethyl & Laboratories, A301-910A-T)	1:200 rabbit
Anti-MERVL (Hang zhou HuaAn Biotechnology Co.,Ltd)	1:100 rabbit
anti-ZSCAN4 (Abcam, ab106646)	1:500 rabbit

Table S3. RT-qPCR Primers Sequences

Name	Sequence (5' to 3')
Oct4-forward	GCTTGGGCTAGAGAAGGATGTG
Oct4-reverse	TGGCGCCGGTTACAGAAC
Sox2-forward	GCGGCGGAAAACCAAGA
Sox2-reverse	CCGGGAAGCGTGTACTTATCC
Nanog-forward	CTTTCACCTATTAAGGTGCTTGC
Nanog-reverse	TGGCATCGGTTCATCATGGTAC
Nelfa-forward	TGCTAGTGGACACAGTGTTCGA
Nelfa-reverse	TTGAAGCGTGTCCACTGGCC
Zscan4-forward	AAATGCCTTATGTCTGTTCCCTATG
Zscan4-reverse	TGTGGTAATTCCTCAGGTGACGAT
Zscan4c-forward	GATTATTGGCCACAGGACAAG
Zscan4c-reverse	TCAGGGTGCTGTTCTTTCTG
Dppa2-forward	TCAACGAGAACCAATCTGAGGA
Dppa2-reverse	GCGTAGCGTAGTCTGTGTTTG
Eif1a-like-forward	AACAGGCGCAGAGGTAAAAA
Eif1a-like-reverse	CTTATATGGCACAGCCTCCT
Dux-coding-forward	AAAGGAAGAGCATGTGCCAGC
Dux-coding-reverse	GCAGTAAGCTGTCCTGGGAAC
Gata2-forward	CCAGCAAATCCAAGAAGAGC
Gata2-reverse	AGACTGGAGGAAGGGTGGAT
Tcstv1-forward	TGAACCCTGATGCCTGCTAAGACT
Tcstv1-reverse	AGATGGCTGCAAAGACACAACTGC

Tcstv3-forward	AGAAAGGGCTGGAACTTGTGACCT
Tcstv3-reverse	AAAGCTCTTTGAAGCCATGCCCAG
Bcl2-forward	CGGGAGAACAGGGTATGAT
Bcl2-reverse	ATTGGGTTGCTCTCAGGCT
Gata4-forward	TTCCTCTCCCAGGAACATCAAA
Gata4-reverse	GCTGCACAACTGGGCTCTACTT
Gata6-forward	TGCTGGAAATTGCAACAAACC
Gata6-reverse	GTCACGTGGTACAGGCGTCA
Sox17-forward	GTCAACGCCTTCCAAGACTTG
Sox17-reverse	GTAAAGGTGAAAGGCGAGGTG
Hand1-forward	TCAAAAAGACGGATGGTGGT
Hand1-reverse	GCGCCCTTTAATCCTCTTCT
Brachyury (T) -forward	GAACCTCGGATTCACATCGT
Brachyury (T) -reverse	TTCTTTGGCATCAAGGAAGG
Evx1-forward	CCAGTGACCAGATGCGCCGATAC
Evx1-reverse	TCCTTCATGCGCCGGTTCT
Nestin-forward	CTCGAGCAGGAAGTGGTAGG
Nestin-reverse	TTGGGACCAGGGACTGTTAG
Sox1-forward	GGCCGAGTGGAAGGTCATGT
Sox1-reverse	TCCGGGTGTTCCTTCATGTG
Pax6-forward	GCAGATGCAAAAGTCCAGGTG
Pax6-reverse	CAGGTTGCGAAGAACTCTGTTT
Cdx2-forward	CCTGCGACAAGGGCTTGTTTAG
Cdx2-reverse	TCCCGACTTCCCTTCACCATAC
Gapdh-forward	ATGGTGAAGGTCGGTGTGAAC
Gapdh-reverse	TCGCTCCTGGAAGATGGTGATG

The uncropped western blots

Figure 1C

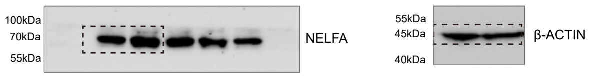

Figure 1F

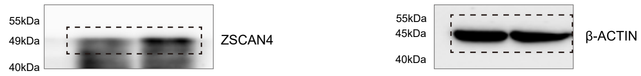

Figure 2C

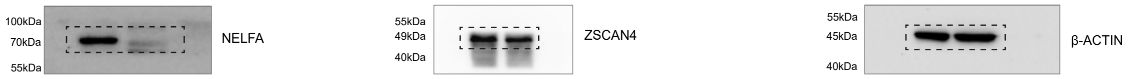

Figure 3C

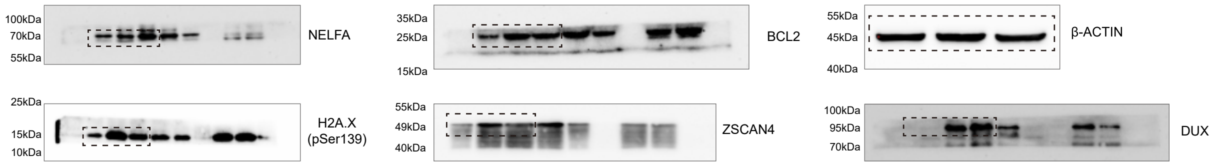

Figure 4G

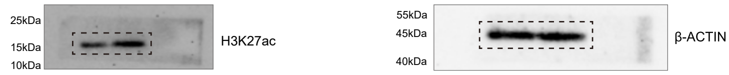

Figure 5C

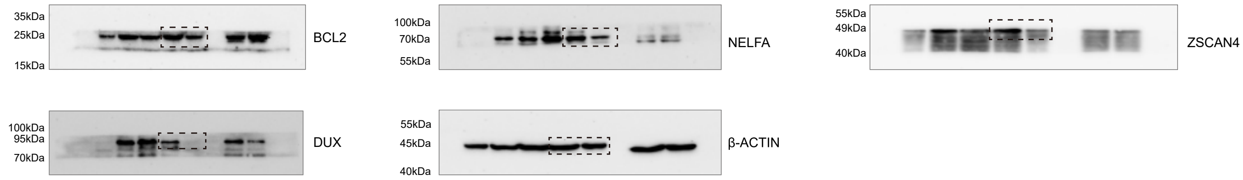

Figure 6D

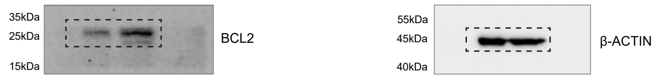

Figure 7E

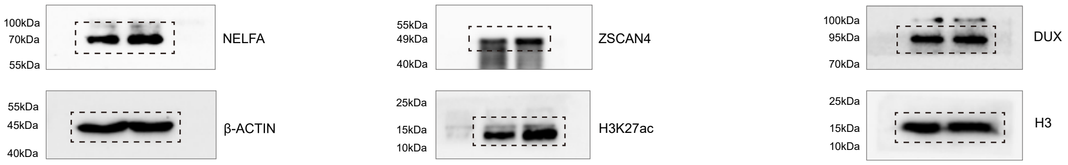

Supplement: Supplementary file 1 — Data S1. Supporting Information. [file CPR-57-e13534-s001.pdf]
